# Network Hubs Buffer Environmental Variation in Saccharomyces cerevisiae

**DOI:** 10.1371/journal.pbio.0060264

**Published:** 2008-11-04

**Authors:** Sasha F Levy, Mark L Siegal

**Affiliations:** Center for Genomics and Systems Biology, Department of Biology, New York University, New York, New York, United States of America; Johns Hopkins University, United States of America

## Abstract

Regulatory and developmental systems produce phenotypes that are robust to environmental and genetic variation. A gene product that normally contributes to this robustness is termed a phenotypic capacitor. When a phenotypic capacitor fails, for example when challenged by a harsh environment or mutation, the system becomes less robust and thus produces greater phenotypic variation. A functional phenotypic capacitor provides a mechanism by which hidden polymorphism can accumulate, whereas its failure provides a mechanism by which evolutionary change might be promoted. The primary example to date of a phenotypic capacitor is Hsp90, a molecular chaperone that targets a large set of signal transduction proteins. In both *Drosophila* and *Arabidopsis*, compromised Hsp90 function results in pleiotropic phenotypic effects dependent on the underlying genotype. For some traits, Hsp90 also appears to buffer stochastic variation, yet the relationship between environmental and genetic buffering remains an important unresolved question. We previously used simulations of knockout mutations in transcriptional networks to predict that many gene products would act as phenotypic capacitors. To test this prediction, we use high-throughput morphological phenotyping of individual yeast cells from single-gene deletion strains to identify gene products that buffer environmental variation in Saccharomyces cerevisiae. We find more than 300 gene products that, when absent, increase morphological variation. Overrepresented among these capacitors are gene products that control chromosome organization and DNA integrity, RNA elongation, protein modification, cell cycle, and response to stimuli such as stress. Capacitors have a high number of synthetic-lethal interactions but knockouts of these genes do not tend to cause severe decreases in growth rate. Each capacitor can be classified based on whether or not it is encoded by a gene with a paralog in the genome. Capacitors with a duplicate are highly connected in the protein–protein interaction network and show considerable divergence in expression from their paralogs. In contrast, capacitors encoded by singleton genes are part of highly interconnected protein clusters whose other members also tend to affect phenotypic variability or fitness. These results suggest that buffering and release of variation is a widespread phenomenon that is caused by incomplete functional redundancy at multiple levels in the genetic architecture.

## Author Summary

Most species maintain abundant genetic variation and experience a wide range of environmental conditions, yet phenotypic differences between individuals are usually small. This phenomenon, known as phenotypic robustness, presents an apparent contradiction: if biological systems are so resistant to variation, how do they diverge and adapt through evolutionary time? Here, we address this question by investigating the molecular mechanisms that underlie phenotypic robustness and how these mechanisms can be broken to produce phenotypic heterogeneity. We identify genes that contribute to phenotypic robustness in yeast by analyzing the variance of morphological phenotypes in a comprehensive collection of single-gene knockout strains. We find that ∼5% of yeast genes break phenotypic robustness when knocked out. The products of these genes tend to be involved in critical cellular processes, including maintaining DNA stability, processing RNA, modifying proteins, and responding to stressful environments. These genes tend to interact genetically with a large number of other genes, and their products tend to interact physically with a large number of other gene products. Our results suggest that loss of phenotypic robustness might be a common phenomenon during evolution that occurs when cellular networks are disrupted.

## Abstract 3

A genome-wide screen in*Saccharomyces cerevisiae* identifies over 300 gene products that buffer environmental variation--dubbed phenotypic capacitors--and function as hubs in protein-protein and synthetic-lethal interaction networks.

## Introduction

The relationship between genotype and phenotype is a central concern of many fields, from developmental biology to human genetics to evolutionary biology. Although in most cases this relationship is poorly understood, some general properties do seem to be shared across diverse systems. Chief among these is robustness to genetic and environmental variation [1]. That is, most species maintain abundant genetic variation and experience a wide range of environmental conditions, yet phenotypic variation is relatively low [2]. Because of its ubiquity, phenotypic robustness, also termed canalization or buffering, is worthy of study in its own right [3]. It also presents an apparent contradiction: if biological systems are so robust, how do they diverge and adapt through evolutionary time?

The contradiction might be resolved if the robustness itself were to be modulated by particular mutations or environmental conditions. The robust system would accumulate conditionally neutral, or “cryptic,” genetic variation. A genetic or environmental perturbation that impaired the system's robustness would then reveal the cryptic variation in the form of greater phenotypic diversity. The modulation of robustness would not only allow evolutionary divergence, but it might also accelerate it relative to the slow, step-wise fixation of fitness-increasing alleles that is normally considered within the Neodarwinian paradigm [3–5]. It is therefore essential to investigate mechanisms that contribute to the robustness of biological systems, and to understand how such mechanisms determine the phenotypic effects of different sources of variation.

A model for the buffering and release of variation is provided by the molecular chaperone Hsp90, which targets a large set of signal transduction proteins. In both *Drosophila* and *Arabidopsis*, compromised Hsp90 function results in diverse morphological changes that exhibit strong dependence on the genetic background [6,7]. This implies that Hsp90 normally contributes to phenotypic robustness to genetic variation. Because Hsp90 function allows stores of genetic variation to build up, and Hsp90 impairment releases this variation to have phenotypic effects, it is termed a “phenotypic capacitor” [7].

Controversy surrounds the evolutionary relevance of Hsp90-mediated capacitance and any similar mechanisms that might exist. One issue is whether any fraction of the phenotypic variation revealed by an impaired capacitor is adaptive, or instead whether the variants consist entirely of hopeful, but ultimately unfit, monsters [8,9]. The major morphological defects seen originally in flies [7] support the latter conclusion, yet selection for one such defect did not cause correlated fitness costs, suggesting that the pleiotropic effects of Hsp90 impairment are modular and not unconditionally deleterious [10]. The variation seen in *Arabidopsis* also supports the potential adaptive value of capacitance, in that this variation is considerably less monstrous than that seen in flies [6]. A more systematic analysis of the phenotypic and fitness effects of capacitor impairment is needed to resolve this issue.

A second debate concerns the ultimate evolutionary reason that capacitance exists. One view is that the ability to modulate evolvability is itself an adaptive trait, and that natural selection has therefore favored capacitor function [11]. This view generally meets with great skepticism, as do similar views on the evolutionary benefits of mechanisms that alter mutation or recombination rates [9]. Nonetheless, a population-genetic model has shown that an allele that modifies the rate of revelation of cryptic genetic variation can invade a population under a realistic range of parameter values [12,13]. Although adaptive evolution of capacitance therefore remains a formal possibility, many favor an alternative view that considers capacitance a side effect of other selected properties. One possibility is that natural selection favors mechanisms that buffer against environmental variation, with environmental variation taken to mean both large macro-environmental differences and stochastic fluctuations in the external micro-environment or internal cellular environment. The buffering of genetic variation then results from a hypothesized mechanistic congruence between the impacts of allelic variation and environmental variation on regulatory networks [14]. Another possibility is that regulatory networks inherently attenuate variation because they contain thresholds and other nonlinearities that allow them to respond properly to internal or external cues [8,15]. Indeed, our own simulations of evolving regulatory networks predicted that many gene products should act as phenotypic capacitors, contributing to phenotypic robustness when present and producing greater phenotypic diversity when absent [16].

The above considerations motivate the development of an experimental system in which many phenotypes can be precisely measured in many individuals, multiple gene products can be screened for capacitor function, and sources of variation can be precisely controlled and partitioned. Here we present such a system, using single-cell morphological phenotypes in the yeast *S. cerevisiae.* We focus here on the robustness of these phenotypes to environmental variation caused by stochastic fluctuations in a constant macro-environment. While the study of robustness to environmental variation is critical to understanding the development and physiology of organisms, it also lays the foundation for future work that will rigorously test the congruence between mechanisms of environmental and genetic buffering and that will investigate the impact of capacitors on evolutionary trajectories.

**Figure 1:**
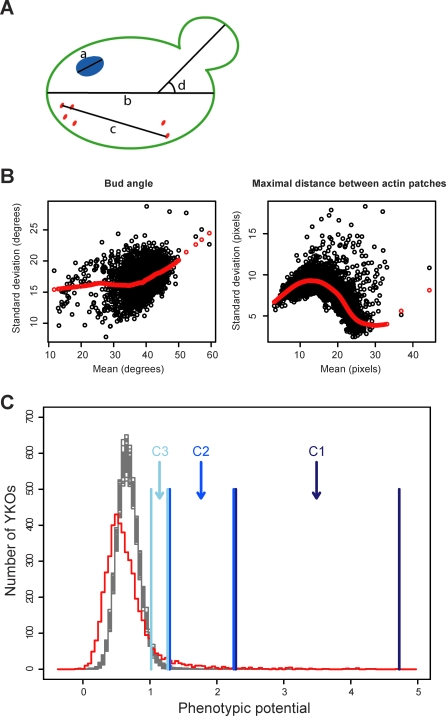
Genome-Wide Screen for Phenotypic Capacitors in S. cerevisiae (A) Schematic of some quantitative phenotypes in a budded cell: (a) long axis length of the mother nucleus, (b) long axis length in the mother cell, (c) maximal distance between actin patches, (d) bud angle. A full list of phenotypes and their descriptions is reported in Ohya et al. [17]. (B) Scatter plots of means and standard deviations for 4,718 YKO strains for two phenotypes clearly indicate that variance depends on mean in a nontrivial, phenotype-specific manner. Black circles represent individual YKOs and red circles represent the lowess curve fit. (C) Histograms of actual (red) and 100 randomized (grey) phenotypic potential scores. Shown are the ranges of the three phenotypic capacitor classes (C1, C2, C3).

To identify gene products that contribute to robustness to environmental variation, we take advantage of data from high-dimensional quantitative morphological phenotyping of 4,718 haploid yeast single-gene knockout (YKO) strains [17]. Phenotyping was performed by growing cells in rich media to logarithmic growth phase and triply staining them for the cell surface, actin cytoskeleton, and nuclear DNA. Digital micrographs of ∼200 cells per strain were processed using automated image analysis [17], yielding means and variances for 220 diverse quantitative phenotypes for each YKO (Figure 1A). The phenotypes include measures of the size and shape of mother and bud cells and their nuclei, the number and size of actin patches, the position of nuclei or actin patches in reference to other cell landmarks, and relationships between the mother and bud, such as the bud angle (for a complete list, see [17]).

Using these data we identify more than 300 gene products required for robustness to environmental variation. We find that these capacitors are involved in a number of critical cellular processes and that they are highly connected, in terms of both physical and genetic interaction networks. Despite this centrality, capacitor deletions result on average in decreases in growth rate that could allow these mutants to persist for many generations in the presence of wild-type cells, suggesting that capacitor impairment need not produce unfit monsters. Capacitors encoded by a member of a duplicate gene pair differ in their functional and network properties from those encoded by singletons, suggesting that these two classes of capacitors are likely to buffer environmental variation by different mechanisms.

## Results

We used reported means and variances from quantitative morphological phenotyping of 4,718 haploid YKO strains [17] to identify phenotypic capacitors. Our working definition of a capacitor is a gene product that causes high variance in multiple nonredundant phenotypes when deleted. To identify gene products that meet this criterion, three obstacles must be overcome: (1) a measure of variance that is not dependent on the mean must be generated so as not to confound changes in variance with changes in mean phenotype [18]; (2) biologically or physically redundant phenotypes must be eliminated (dimensional reduction); and (3) a robust score for the overall variance in multiple phenotypes must be generated.

We addressed the first obstacle by plotting, for each phenotype, the standard deviation versus the mean of each YKO, fitting a lowess (locally weighted) regression to each plot, and calculating the residual distance of each point from the regression (Figures 1B and S1). These residuals of standard deviation, which represent a measure of variance controlled for the mean, were standardized to weight each phenotype equally.

For dimensional reduction, we used partitioning around medoids (PAM), a robust variant of k-means clustering. By analyzing the average silhouette width, a measure of separation of clusters, we estimated that the 220 original phenotypes can be reduced to 70 representative phenotypes (“medoids”) (Figure S2).

To generate a single measure of the overall phenotypic variance when a gene is deleted, we averaged the top 35 (of 70) residuals of standard deviation for each YKO (Table S1). This score, which we term phenotypic potential, was extremely robust to even large changes in the clustering and averaging procedure and did not appear to be biased by potential edge effects in the lowess procedure (Figures S3–S6).

To identify genes with significantly higher phenotypic potential scores than expected by chance, we compared the distribution of scores of all YKOs to distributions generated when values within each phenotype are first permuted. Based on this permutation analysis, the 502 genes with the highest phenotypic potential were identified as putative phenotypic capacitors with an estimated false discovery rate (FDR) of 0.34 (Figure 1C). This FDR maximizes the estimated number of true positives (Figure S7 and see Materials and Methods). We thus estimate that 333 genes out of the total 502 identified are true positives, with a higher phenotypic potential than would be predicted by chance. For downstream analysis, the top 502 genes were separated into three classes. The top 60 high-confidence genes (FDR = 0), the next 206 mid-confidence genes (FDR cutoff = 0.10), and the next 266 low-confidence genes (FDR cutoff = 0.34) are termed “C1,” C2,” and “C3,” respectively.

### Validation of Phenotypic Capacitors

**Figure 2:**
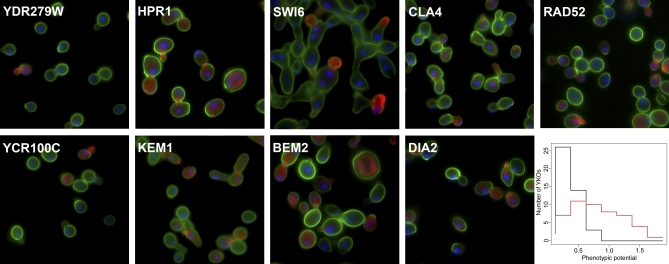
Phenotypic Capacitor YKOs Have Highly Variable and Distinct Phenotypes Representative micrographs from examples of putative phenotypic capacitor (HPR1, KEM1, SWI6, BEM2, CLA4, DIA2, RAD52) and control (YDR279W and YCR100C) strains are shown. Cells are stained with FITC-concanavalin A (green), rhodamine phalloidin (red), and DAPI (blue). Lower right: Histograms of the phenotypic potentials of control (black) and phenotypic capacitor (red) YKOs of haploid-converted heterozygous diploid strains.

We validated our identification of capacitors by repeating the phenotyping of 50 C1 strains and 50 control strains in our lab. Haploid mutants in the YKO library used for the original phenotyping were passaged for an unknown number of generations. Because some knockouts might increase mutation rates and thereby cause phenotypic variability that is not associated with loss of environmental buffering, we used instead a haploid-convertible heterozygous diploid YKO library [19] and kept the number of generations between sporulation and fixation to a maximum of ∼50. C1 strains exhibited more phenotypic variability than control strains (Figures 2 and S8). Phenotypes also appeared to be highly heterogeneous among C1 strains, suggesting that knockouts are not all disrupting a small number of high-level processes that result in a limited set of phenotypes. With the phenotype means and standard deviations from only the 100 haploid-converted strains, we repeated the calculation of phenotypic potential as described above, using the 70 already identified phenotypic medoids (Figure 2, lower right). Our expectation was that this repeated analysis would have reduced power because the proportionally large number of C1 strains that exhibit high variance would bias the lowess regression to decrease the magnitude of residuals. Even with this limitation, we found the phenotypic potential scores to be clearly higher in the C1 strains (*p* < 9.8 × 10^−10^, Wilcoxon-Mann-Whitney test). For a less conservative measure of phenotypic potential, we sampled data from capacitor and control strains in rough proportion to their numbers in the original analysis (five to 42), calculated scores on 1,000 repeated samplings, and averaged across all the 1,000 samples to calculate final phenotypic potentials (Figure S9). While this analysis still results in a conservative bias because of a reduction of residuals at the edges of the lowess regression due to fewer data points, capacitors exhibit ∼2-fold higher phenotypic potentials than controls (*p* < 2.7 × 10^−10^, Wilcoxon-Mann-Whitney test). A notable exception is RAD27, which has a lower phenotypic potential than all but three of the control strains in the more conservative first analysis and all but nine in the less conservative sampling analysis. RAD27 deletion causes a strong spontaneous mutator phenotype [20], which suggests that the high phenotypic variability observed in the original YKO might have been due to mutation accumulation. We also sampled ten capacitors in the C2 or C3 classes and these too exhibited significantly higher phenotypic potential scores than control strains (*p* < 0.002, Wilcoxon-Mann-Whitney test).

As additional validation, we looked for evidence in the literature of capacitors causing increased cell-to-cell variability. Indeed, knockouts of the capacitors CCR4, CLN3, and SWI6 have each been shown to result in increased variability in diploid cell size in liquid media [21]. Knockout of CCR4 also causes irregular colony morphology on solid medium, a finding consistent with increased cell-to-cell variation [22]. Two other members of the CCR4-NOT core complex, NOT5 and POP2, are identified as capacitors in our screen suggesting that disruption of this transcriptional regulatory complex is likely to result in cellular heterogeneity. Additionally, two of three knockouts that increase intrinsic expression noise of the PHO5 promoter are the capacitors ARP8 and SNF6 [23]. Lastly, knockout of the capacitor FUS3 has been shown to increase cell-to-cell variation in response to a pheromone signal [24].

### Phenotypic Capacitors Are Enriched in Several Gene Ontology Categories

We next investigated the entire set of 502 capacitors for enrichment in Gene Ontology (GO; http://www.geneontology.org/) process terms (Table S2). Phenotypic capacitors are highly enriched in numerous processes, most of which can be broadly categorized into DNA maintenance and organization, cell cycle and cell organization, response to stimuli such as stress, RNA elongation, or protein modification. This diverse set of enriched terms is likely to represent processes that can result in broad cellular changes when disrupted.

Specifically, capacitors contain 123 of 565 ORFs annotated with “chromosome organization and biogenesis” and 80 of 275 ORFs annotated with its daughter term, “telomere organization and biogenesis” (*p* < 2.9 × 10^−27^ and *p* < 2.1 × 10^−25^, respectively, hypergeometric distribution with Bonferroni correction). These include all genes annotated with recombinase activity, the entire telomerase holoenzyme complex, the entire RecQ helicase-Topo III complex, all but one gene from the homologous recombination module, all genes involved in postreplication repair, the entire CTF18m complex involved in sister chromatid cohesion and DNA-replication check-point signaling, the entire MMS22m module thought to be involved in double strand break repair, and both genes of the HEX3m module [25]. Notably, however, other modules that are likely to cause DNA instability are absent, including the nucleotide excision repair module, the DNA damage checkpoint module RAD9m, the MUS81m module involved in cleaving branched DNA, and the TOF1m module involved in promoting sister chromatid cohesion to repair DNA damage [25].

Capacitors also include 28 transcriptional regulators; numerous gene products involved in global mRNA production, including all members of the carboxy-terminal domain protein kinase complex; most members of the THO complex, which is thought to couple transcriptional elongation with mRNA metabolism and export; numerous nuclear pore-associated proteins, including both members of the mRNA export SAC3/THP1 complex; at least seven gene products involved in mRNA splicing; approximately half of the gene products annotated to the cytoplasmic mRNA processing (P) body; genes involved in protein transport and degradation, including three members of the Golgi-localized alpha-1,6-mannosyltransferase complex and eight gene products involved in vacuolar acidification; and genes involved in the control of actin (nine genes) and microtubule (12 genes) organization and in bud emergence or selection*.*

### Phenotypic Capacitors Are Likely to Be Network Hubs

Protein–protein interaction (PPI) and synthetic lethal interaction (SLI) networks have a small number of highly connected nodes (hubs) and many more poorly connected nodes [26,27]. In PPI networks, deletion of a hub is more likely to be lethal than deletion of other nodes [26]. This finding suggests that genetic properties are, at least in part, traceable to global network architecture. Our numerical simulations of transcriptional networks implied that robustness is likely to be an emergent property of complex networks, and that in some cases network architecture constrains functional and evolutionary properties [15,16,28]. Others have predicted that SLIs are crucial to understanding buffering [29] or that phenotypic capacitors are likely to be hubs [30,31]. Thus, we asked here if a gene's phenotypic potential is traceable to network position.

**Figure 3:**
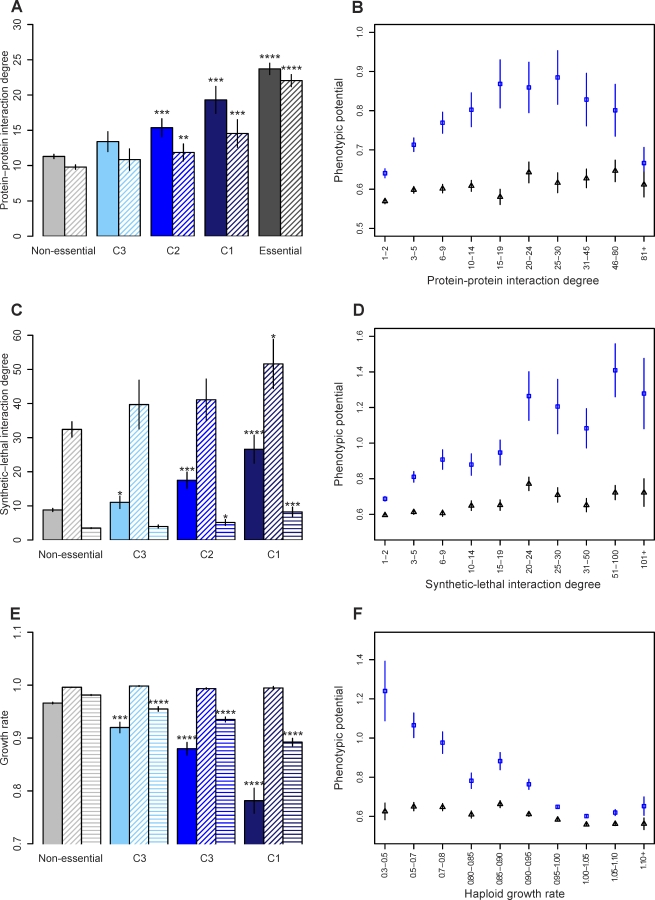
Network and Dispensability Characteristics of Phenotypic Capacitors (A) The average number of physical interactions of high (C1, dark blue), mid (C2, blue), and low (C3, light blue) confidence phenotypic capacitors versus all nonessential (light grey) or essential (dark grey) genes using all physical interactions in the literature-curated network of the BioGRID (solid bars) or only affinity-capture mass spectrometry interactions (diagonal stripes). (B) The average phenotypic potential of all nonessential genes (blue squares) or nonessential genes that have not been classified as a phenotypic capacitor (black triangles) binned by their number of affinity-capture mass spectrometry PPIs. (C) The average number of SLIs using all interactions annotated in the literature curated BioGRID network (solid bars), interactions derived only from SGA experiments considering only genes used as bait (diagonal stripes), and interactions derived only from SGA experiments considering only genes not used as bait (horizontal stripes). (D) The average phenotypic potential of all nonessential (blue squares) or nonessential noncapacitor (black triangles) genes binned by their number of literature-citation SLIs. (E) The average growth rates for haploid KO (solid bars), heterozygous diploid KO (diagonal stripes), and homozygous diploid KO (horizontal stripes) strains. Values are relative to wild type. (F) The average phenotypic potential of all nonessential (blue squares) or nonessential noncapacitor (black triangles) genes binned by their haploid growth rates. Error bars represent the standard error of the mean. All *p*-values are a comparison to nonessential genes, Wilcoxon-Mann-Whitney test: *, *p* < 0.05; **, *p* < 0.001; ***, *p* < 1 × 10^−5^; ****, *p* < 1 × 10^−10^.

First, we used networks derived both from curated literature citations [32] and from the nearly complete set of affinity-capture mass spectrometry interactions [33,34] to determine if the average PPI degree (number of interactions) of capacitors is different than that of other genes. We found that capacitors have more physical interactions than other nonessential gene products but fewer than essential gene products (Figure 3A). Because capacitors are enriched in select GO categories, one potential explanation for the high degree of capacitors is that they fall into GO categories whose members tend to be highly connected. However, in most cases, capacitors have significantly more interactions than GO-matched nonessential genes (Figure S10). Exceptions to this rule appear to occur mostly in GO categories where essential and nonessential genes do not differ in PPI degree.

A plot of phenotypic potential versus binned PPI degree shows that gene products with a higher connectivity have on average a higher phenotypic potential (Figure 3B); this relationship is almost entirely explainable by an increased proportion of capacitors in the highly connected bins (Figure S11). Interestingly, the proportion of capacitors precipitously drops at PPI degrees above 30, with similar proportions in the highest (>80 PPI) and lowest (<3 PPI) bins. The vast majority of genes in this highest bin have a duplicate in the genome, including 28 ribosomal proteins, three histones, and, of particular note, the homologues of Hsp90: HSP82 and HSC82. While surprising, the finding that Hsp90 homologues do not act as capacitors in S. cerevisiae according to our definition is consistent with a previous study that found that Hsp90 activity, not impairment, allowed new mutations to have immediate phenotypic consequence [35]. However, we do find a homologue of the chaperone Hsp70, SSE1, to be a capacitor in yeast, consistent with studies on other Hsp70 family members [36].

Next, we used all SLIs from curated literature citations [32] and, again, find that capacitors have a higher degree than other nonessential genes (Figure 3C, solid bars). Because genome-scale SLI assays have only been completed for a subset of genes (∼267 used as bait in synthetic genetic array [SGA] experiments), one potential explanation for the high SLI degree of capacitors is that they were more likely to be used as bait. To control for this possibility, we generated a subnetwork derived only from SGA experiments and divide genes in this network into those that had been used as bait and those that had not. For each class, we found that capacitors have higher SLI degree than other nonessential genes (Figure 3C, striped bars). The higher SLI degree of capacitors is maintained even when the number of physical interactions of a gene is controlled for (Figure S12) or when capacitors are compared to other nonessential genes within GO categories (Figure S13). A plot of phenotypic potential versus binned SLI degree (Figure 3D) shows that genes with a higher connectivity have on average a higher phenotypic potential; this relationship is almost entirely explainable by an increased proportion of capacitors in the highly connected bins (Figure S11).

### Effects of Phenotypic Capacitor Knockouts on Growth Rate

We have shown that capacitors are likely to be PPI and/or SLI hubs, that they might be less likely to be completely functionally redundant, and that they are involved in a number of central processes in the cell. At this point, one might ask if disruption of capacitor function has too drastic of an effect on fitness to play a role in adaptation. To investigate this possibility, we asked if capacitors are less dispensable than other nonessential genes by comparing the growth rates of haploid [37] or diploid [38] mutants. Whereas capacitors appear to have no effect on growth rate in the heterozygous diploid, they are less dispensable in haploid and homozygous diploid knockouts, with a larger rate decrease seen in the haploid (Figure 3E). This effect is maintained even when we controlled for the PPI degree (Figure S12) or when capacitors were compared to other nonessential genes within GO categories (Figure S14). Gene knockouts that result in drastically reduced growth rates (less than 70% of wild-type) on average have higher phenotypic potentials (Figure 3F); this relationship is entirely explainable by an increased proportion of capacitors that cause low growth rates (Figure S11). However, in most cases, capacitors cause decreases in growth rate that are not as severe, with 79% and 95% of capacitor YKOs having a growth rate exceeding 0.80 of wild-type in the haploid and homozygous diploid, respectively. Indeed, 124 capacitor YKOs have an increased growth rate over the wild type.

### Duplicate and Singleton Capacitors Have Different Modes of Functional Redundancy

**Figure 4:**
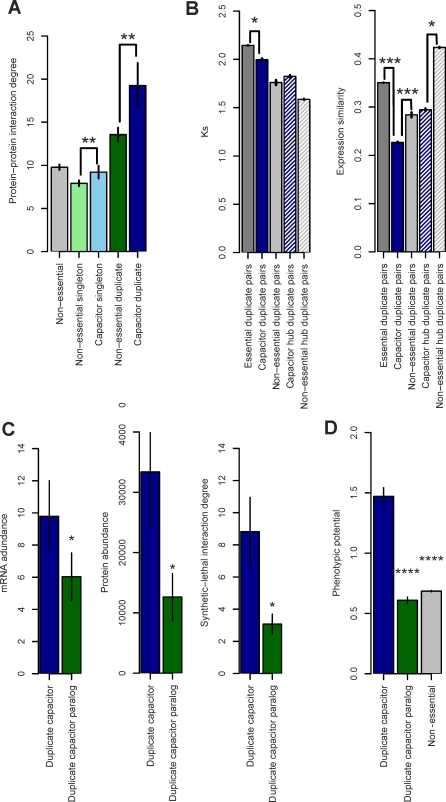
Characteristics of Capacitor Duplicates (A) The average number of affinity-capture mass spectrometry interactions of all nonessential genes (grey), all nonessential singletons (light green), capacitor singletons (light blue), all nonessential duplicates (dark green), and capacitor duplicates (dark blue). (B) Ks (left) and the expression similarity (right) between duplicate pairs that contain at least one essential gene (dark grey), at least one capacitor (solid dark blue), only nonessential noncapacitor genes (solid light grey), at least one gene product with over 19 PPIs annotated in the literature-curated BioGRID network and at least one capacitor (dark blue stripes), and at least one gene product with over 19 PPIs and no capacitors or essential genes (light grey stripes). (C) The mRNA abundance (left), protein abundance (middle), and SLI degree (right) of capacitors with only one paralog in the genome (blue) and their paralogs (green). (D) The phenotypic potential of capacitors with only one paralog in the genome (blue), their paralogs (green), and all nonessential genes (grey). Error bars represent the standard error of the mean. Unless otherwise marked, *p*-values are a comparison to duplicate capacitors, Wilcoxon-Mann-Whitney test: *, *p* < 0.05; **, *p* < 0.001; ***, *p* < 1 × 10^−5^; ****, *p* < 1 × 10^−10^.

The finding that many of the most highly connected PPI hubs are duplicates but not capacitors (Figure 3B) suggests a relationship between functional redundancy and buffering. We investigated this further by asking how capacitor genes distribute among the 1,425 unambiguous duplicates and 2,375 unambiguous singletons that have been identified in the yeast genome [39–41]. We found that capacitor genes are more likely to be singletons than other nonessential genes (Table 1, *p* < 0.026, G-test). Capacitor singletons and capacitor duplicates tend to be enriched in different GO process categories. Capacitor singletons are enriched in the categories of DNA maintenance and organization, response to stimuli, and RNA transcription and localization (Table S3). Capacitor duplicates, while more heterogeneous overall, tend to be most enriched in the categories of protein metabolism and endocytosis. We next investigated if capacitor singletons differ from capacitor duplicates in any network or dispensability properties. Both duplicate and singleton capacitor genes tend to have a high number of SLIs and to cause decreases in growth rate when knocked out in the haploid or homozygous diploid (Figure S15). However, only capacitor duplicates tend to be PPI hubs (Figure 4A).

**Table 1:**
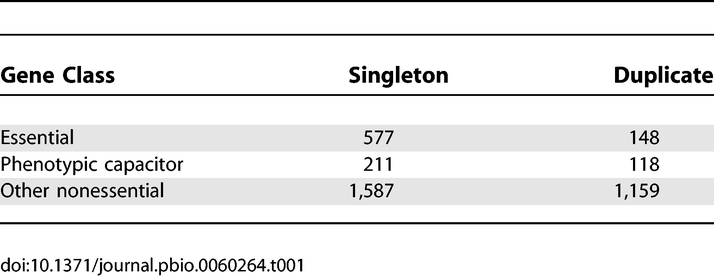
Number of Singletons or Duplicates in Different Gene Classes

The above finding presents an apparent contradiction: high PPI degree is strongly associated with capacitor identity in duplicates (Figure 4A) yet many highly connected duplicates are not capacitors (Figure 3B). The resolution of this contradiction appears to be that capacitor duplicate pairs are older and have less functional redundancy than other hub duplicate pairs. Using the synonymous substitution rate (Ks) between members of a duplicate pair as a rough estimate of age of the duplication event [39,42], we found that duplicate pairs that contain at least one capacitor are on average less ancient than duplicate pairs that contain at least one essential gene and appear to be more ancient than duplicate pairs that contain only nonessential noncapacitor genes (Figure 4B). Because substitution rates correlate negatively with expression level of a gene [43,44], one explanation for the differences in Ks is that duplicates that contain at least one capacitor have a different distribution of expression levels than other duplicate pairs. However, we found the pattern persists even when we control for mRNA expression level (Figure S16). The average age difference between capacitor duplicate pairs and nonessential duplicate pairs appears to be due to recent duplicates (Ks < 1), of which there are fewer capacitor duplicate pairs than nonessential noncapacitor duplicate pairs (*p* < 0.002, G-test, Figure S17). The same relation holds when comparing only hub duplicate pairs, which we defined as duplicate pairs where at least one paralog has a PPI degree ≥ 20. To calculate this difference, we separated hub duplicate pairs into those that contain at least one capacitor and those that do not. The average PPI degree of gene products in these two categories is approximately the same (*p* = 0.53, Wilcoxon-Mann-Whitney test). Comparing these two categories, we found that hub capacitor duplicates appear to be older on average due to an absence of recent duplication events. The greater average age of capacitor duplicate pairs raises the possibility that they are predominantly ohnologs, tracing their origin to the whole genome duplication in yeast ∼100 million years ago [45]. However, we did not find ohnologs to be overrepresented among capacitor duplicates relative to noncapacitor duplicates (*p* = 0.47, G-test). The greater synonymous-site divergence of capacitor duplicate pairs does not appear to be matched by greater divergence, as measured by the nonsynonymous substitution rate (Ka). For this comparison, we restricted our analysis to ohnologs to control for differences in duplication time that might introduce errors in Ka/Ks. Capacitor ohnologs have Ka values that are not significantly different from nonessential ohnologs, whereas essential ohnologs show marginally greater divergence (Figure S18).

A more striking difference between capacitor duplicate pairs and other duplicate pairs was evident in the correlations in expression between paralogs across many experimental conditions [39]. Duplicate pairs that contain at least one capacitor have a lower expression similarity on average than duplicate pairs that contain an essential gene or noncapacitor nonessential duplicate pairs (Figure 4B). Hub duplicate pairs that contain at least one capacitor also have a lower expression similarity than other nonessential hub duplicate pairs. These findings suggest that capacitor duplicate pairs are less functionally redundant than other duplicate pairs. To further dissect this difference in correlated expression, we examined the 64 capacitor-containing duplicate pairs in which both members of the pair have only one paralog in the genome. From these pairs, we excluded two pairs where both copies encode capacitors (the ribosomal proteins RPL8A and RPL8B and the mannotransferases HOC1 and OCH1) and two pairs where a capacitor gene is paired with an essential gene (the cyclins CDH1 and CDC20 and the UDP-glucose phosphorylases YHL012W and UGP1), yielding 60 capacitor genes paired with a nonessential noncapacitor gene. Comparing only these 60 capacitors to their paralogs, we found that the capacitor is likely to have a higher mRNA and protein abundance than its noncapacitor duplicate (Figure 4C).

Perhaps because of these expression profile differences, the capacitor gene has on average approximately three times as many SLIs as its paralog (Figure 4C). The higher expression of the capacitor in the pair suggests that perhaps the noncapacitor paralog also has an effect on robustness but a smaller one that did not surpass our threshold for capacitor identification. However, the noncapacitor paralogs do not have elevated phenotypic potentials when compared to all nonessential genes (Figure 4D, *p* = 0.38, Wilcoxon-Mann-Whitney test). Indeed, analysis of noise in protein abundance [46] suggests that the noncapacitor paralogs might be the targets rather than the sources of buffering: nonessential duplicates have on average greater variability in protein abundance than nonessential singletons (*p* < 2.2 × 10^−3^, Wilcoxon-Mann-Whitney test; Figure S15). A partially redundant duplicate (the capacitor gene) might buffer this variability, whereas its deletion might expose this variability at the level of the phenotype.

**Figure 5:**
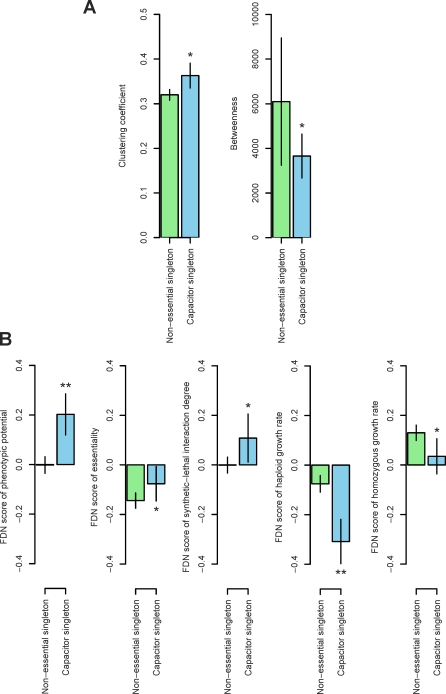
Network characteristics of capacitor singletons (A) The clustering coefficient (left) and betweenness centrality (right) of all nonessential singletons (light green) and capacitor singletons (light blue). (B) Properties of the immediate PPI neighbors of a gene controlled for the degree of that gene (FDN score, see methods) for all nonessential singletons (light green) and capacitor singletons (light blue). From left to right: the phenotypic potential, the number of essential genes, the fraction of SLIs in the literature-curated BioGRID network, the haploid growth rate when deleted, the diploid growth rate when both copies are deleted. Error bars represent the standard error of the mean. Wilcoxon-Mann-Whitney test: *, *p* < 0.05; **, *p* < 0.01.

If one assumes that incomplete functional redundancy is also causing high phenotypic variability in the case of deleted singleton capacitors, understanding the mechanism of this process poses a greater challenge because there are no obvious candidate genes that overlap with singleton function. One hypothesis is that redundancy is achieved not at the level of the single gene as is the case for duplicates, but rather at the level of the protein module. Indeed, many singleton capacitors are part of functionally overlapping modules in the DNA integrity network [25]. To test the hypothesis that this is a more general property of singleton capacitors, we examined further their network characteristics. The clustering coefficient is a measure of local network interconnectivity. Singleton capacitors have on average higher clustering coefficients in the PPI network than all nonessential singletons, suggesting that they are interacting with more tightly knit groups of proteins that might represent functional modules (Figure 5A). Despite their high local connectivity, capacitor singletons occupy less central positions in the overall PPI network than do other nonessential singletons, as measured by betweenness centrality (Figure 5A). This is in stark contrast to capacitor duplicates, which tend to be more central when compared to other nonessential duplicates (Figure S15).

The clustering of singleton capacitors into modules suggests that their other immediate PPI partners, or first-degree neighbors (FDN), might have special network and dispensability characteristics. Because neighbor parameters are not likely to be independent of PPI degree, we first generated FDN scores for each gene product that describe the properties of a gene product's neighbors while controlling for the PPI degree of that gene product (see Materials and Methods). Using these measures, we found that the FDNs of singleton capacitors are more likely to be capacitors, essential genes, or genes with a high SLI degree when compared to all nonessential singletons (Figures 5B and S19). We also found that FDNs of singleton capacitors are more likely to cause decreases in growth rate in haploid or homozygous diploid knockouts. Taken together, these results suggest that singleton capacitors are acting in highly interconnected modules whose other members are likely to disrupt robustness or be deleterious when knocked out.

### Capacitors and Chromosome Stability

Approximately one-fourth of identified capacitors are annotated to be involved in maintaining chromosome stability. While this class of genes still meets all of the criteria for singleton capacitors discussed above, one alternative mechanism to explain the increased phenotypic variability in these YKOs is that they are causing mutations or chromosomal aberrations [47]. Because YKOs that cause drastic increases in the spontaneous mutation rate are relatively well defined and rare (see Protocol S1), we concerned ourselves with YKOs that may be causing chromosomal aberrations, most easily measured by rates of gross chromosomal rearrangements (GCRs) [48]. We found that a high rate of GCRs is neither necessary nor sufficient for high phenotypic potential. For example, focusing on the eight genes in the homologous recombination module [25], we found YKOs that cause both high phenotypic potentials and high GCR rates (RAD50, RAD52, RAD57, XRS2), high phenotypic potentials but relatively low GCR rates (RAD51, RAD54), and low phenotypic potentials but high GCR rates (MRE11) [48]. Additionally, we estimated that measured GCR rates are likely to be too low to explain the phenotypic heterogeneity observed in the haploid-converted strains (see Protocol S1). Thus it is unlikely that GCR events alone explain high phenotypic variance. Another source of genetic variation in these lines could be caused by increased insertion rates of transposable elements such as Ty1 [49,50]. However, we again found that increased Ty1 insertion rates are neither necessary nor sufficient for high phenotypic potential (see Protocol S1). It is also possible that these same disrupted processes are changing the mutational spectrum at microsatellites [51,52]. Although not related to environmental buffering, this last possible mechanism is of great evolutionary interest, given recent findings in yeast [53] and dogs [54] of phenotypic variation driven by tandem-repeat length changes. Another possibility is that DNA stability knockouts are causing changes at the telomere [55,56] or elsewhere that heterogeneously activate cell cycle checkpoints [51,57]. Thus, the mechanism by which this subset of capacitors produces phenotypic variability warrants further study. If, however, mutational mechanisms can be excluded, our findings might provide new insight into human malignancies. Many cancers are associated with mutations in genes involved in DNA stability, including orthologs of the capacitors RAD50 [58], RAD54 [59,60], and YAF9 [61], or with genes with a large number of interactions, such as the transcription factor p53 [62]. Our results suggest that the first advantage mutations in these genes might provide to developing malignancies is phenotypic heterogeneity by way of network-associated loss of robustness.

## Discussion

We show that: (1) there are in excess of 300 phenotypic capacitors of environmental variation in S. cerevisiae; (2) capacitors are highly enriched in GO processes that are likely to result in broad cellular changes when disrupted; (3) capacitors tend to be SLI hubs; (4) most capacitor knockouts result in decreases in growth rate that are not severe; (5) capacitor duplicates tend to be PPI hubs that have undergone a relatively ancient duplication event and diverged in expression from their paralogs; and (6) capacitor singletons tend to be part of highly interconnected protein clusters whose members are likely to disrupt robustness or be deleterious when knocked out. Taken together, these findings strongly suggest that loss of phenotypic robustness is a widespread phenomenon that is a consequence of disrupted physical or genetic-interaction networks.

**Figure 6:**
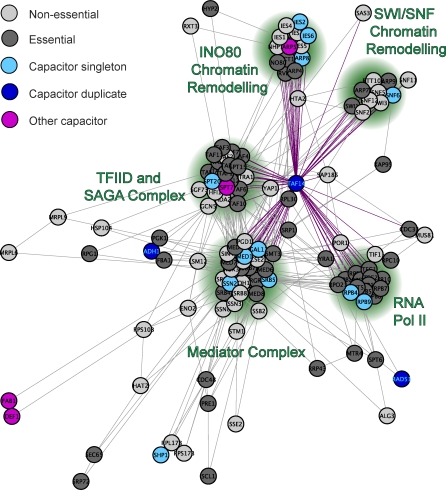
Singleton and duplicate capacitors occupy different locations in the PPI network The differences between singleton and duplicate capacitors are illustrated by the protein-interaction subnetwork surrounding complexes involved in transcriptional activation. Shown is a subnetwork of the affinity-capture mass spectrometry interaction network that was created using all proteins annotated to be in the Mediator complex and their first-degree interaction neighbors. The subnetwork was displayed using Cytoscape (http://www.cytoscape.org/), with nodes placed by spring embedded layout. Nineteen nodes with only one interaction have been removed for clarity. Colors represent nonessential proteins (light grey), essential proteins (dark grey), singleton capacitors (light blue), duplicate capacitors (dark blue), and capacitors that cannot be categorized as singletons or duplicates (purple). Singleton capacitors are likely to be found in highly interconnected complexes such as the Mediator transcriptional coactivation complex, RNA polymerase II, the SAGA and TFIID complexes, and chromatin remodeling complexes. Other members of these complexes tend to be essential genes or capacitors. Duplicate capacitors are highly connected and tend to interact with multiple complexes. Interactions of the duplicate capacitor TAF14 are highlighted in purple.

The mechanism of this disruption appears to be different in cases of duplicate and singleton capacitors, although in both cases incomplete functional redundancy might be causing phenotypic variability when the capacitor is absent (Figure 6). For duplicate capacitors, this partial functional redundancy appears to operate at the level of the paralogous gene pair. Indeed, two properties of duplicate capacitors, high PPI degree and high expression divergence, are found in common with paralogous pairs that are likely to have some redundancy [39]. For singleton capacitors, functional redundancy appears to operate at a higher level of protein modules with partially overlapping function. One common trait shared by both classes of capacitor is a high number of SLIs. The particularly striking finding that nearly 60% of genes with over 100 SLIs are capacitors (Figures 3D and S11) suggests that genetic interactions might be highly predictive of capacitor identity in other organisms [29,30]. A second hallmark of capacitors in other organisms might be the existence of a paralogous gene that has diverged in its regulation, as identified by expression studies or perhaps comparative genomics of *cis*-regulatory regions. With the increasing availability of automated quantitative phenotyping [63], these predictions might soon be possible to test.

While previous studies have suggested that phenotypic capacitor function must be responsive to changes in the environment to influence evolutionary trajectories [6,7], the finding that many capacitors cause decreases in growth rate that are not severe offers another plausible mechanism in yeast: A loss-of-function mutation in a capacitor could be maintained indefinitely in a heterozygous diploid with little or no impact on fitness. Sporulation would be promoted under harsh environmental conditions [64] resulting in haploids with phenotypic variability that is dependent on the (previously cryptic) underlying genotype. Because capacitor loss-of-function usually causes decreases in haploid growth rate that are not severe, these cells could persist for many generations without being out-competed by wild-type counterparts. Some genotypes might provide a selective advantage, which, upon successive rounds of sporulation and mating, could become fixed in the population even in the absence of the capacitor loss-of-function mutation [6,7,16,65]. Alternatively, phenotypic heterogeneity in the absence of genotypic variation might produce epigenetic “persistent” phenotypes with increased fitness in some environments, analogous to those described in models of stochastic phenotype switching [66]. The estimate that approximately 30% of wild S. cerevisiae strains persist for some time as haploids (i.e., are heterothallic) [67,68] suggests that these are plausible mechanisms.

Several challenges remain, however, to understanding mechanistically how phenotypic robustness impacts evolutionary trajectories. One major challenge is characterizing the relationship between environmental and genetic buffering. Phenotypic capacitors identified here buffer environmental variation. Some have predicted that the same mechanisms that buffer environmental sources of variation will also act to buffer genotypic variation [14]. Evidence in support of this hypothesis is mixed and mostly stems from studies of the molecular chaperone Hsp90, a known capacitor of genetic variation in flies and plants [6,7]. Using fluctuating asymmetry in isogenic *Drosophila* lines as a measure of robustness to environmental perturbation, Hsp90 was found to buffer environmental variation in some traits [69] but not others [70]. In *Arabidopsis*, Hsp90 appeared to buffer environmental variation for every trait tested [71]. Results described here provide an experimental system with which to formally test the congruence between the mechanisms of genetic and environmental buffering. Because variability of many quantitative phenotypes can be determined in a high-throughput manner and because we have identified many capacitors of environmental variation, experiments that precisely control and partition different sources of variation can be performed to test if these same gene products contribute to genetic robustness.

Another major challenge is determining if the phenotypic heterogeneity that results from disrupted capacitor function in yeast could be advantageous under natural or artificial selection. One indication that this is likely comes from the finding that disrupted capacitor function might provide an advantage in environments that require invasive growth in yeast. A genetic screen found that mutations in 35 genes can promote haploid invasive growth in the ∑1278 genetic background, 27 of which were considered in our study and 12 of which are phenotypic capacitors [72]. Additionally, we found at least one capacitor YKO that promotes robust haploid invasive growth in the S288C background (SWI6, unpublished data). Stronger evidence comes from a study of stress-sensitive deletion mutants grown in varying concentrations of heavy metals or pro-oxidants [73]. Six deletions, four of which we identify as capacitors (CTR1, CUP5, VMA6, VMA7), resulted in a fitness disadvantage at moderate toxin concentrations but a clear heterogeneity-dependent fitness advantage over the wild-type at high toxin concentrations*.* Also of note, genome-wide examination of genes under positive selection in S. cerevisiae [74] finds nine capacitors out of 72 genes. Because of the relatively short generation time of yeast, it is now possible to formally test if disrupted capacitor function can provide a fitness advantage in some environments.

Although our study has focused on the effects of deletions of nonessential genes, it might be more relevant to common evolutionary trajectories to ask if subtler allelic changes, such as those that result in altered transcription or protein sequence, could cause loss of robustness. Our prediction is that the allelic changes that affect phenotypic heterogeneity are most likely to alter network architecture or dynamics. One possibility is that such mutations would occur in essential genes because, like capacitor genes, they tend to encode highly connected network hubs. Encouraging results come from a recent study that used the progeny from a cross between a yeast lab strain and a wild isolate to map the variances of 35 quantitative phenotypes to 14 quantitative trait loci, one of which is a single nucleotide polymorphism in the essential G-protein alpha subunit GPA1 [75]. The recent availability of a yeast library with decreased expression of essential genes through mRNA perturbation [76] now makes this possible to test on a genome-wide scale.

## Materials and Methods

### Yeast strains.

Haploid convertible diploid BY4743 YKO magic marker deletion strains (*MATa/*α *ura3*Δ*0/ ura3*Δ*0 leu2*Δ*0/leu2*Δ*0 his3*Δ1/ *his3*Δ*1 lys2*Δ*0/LYS+ met15*Δ*0/MET15+ can1*Δ*::LEU2+-MFA1pr-HIS3/CAN1+ xxx::kanMX/XXX+*) were purchased from Open Biosystems.

### General statistics and programming.

All data analysis was performed using the open-source R statistical computing package (http://www.r-project.org/). Lowess regression was performed using the “lowess” function with a smoother span of 0.2 (944 YKOs) and three iterations for the genome-wide analysis (Figure 1C), and with a smoother span of 0.4 (40 YKOs) and five iterations for the repeated analysis (Figure 2). PAM and silhouette plots were performed using the “pam” and “silhouette” functions from the cluster library.

### Dimensional reduction and calculation of phenotypic potential.

A major problem we faced in dealing with a dataset of 220 phenotypes is removing those phenotypes that might be biologically or physically redundant. Principal components analysis, a common means by which to reduce the dimensionality in a matrix (remove redundant phenotypes), transforms the data to a new coordinate system such that the greatest variance of any projection of the data lies on the first coordinate or principal component, the second greatest variance on the second coordinate, etc. This transformation, however, does not preserve the directionality or syntax of the initial dataset because the coordinates are drawn to maximize the overall variance ignoring whether these values are positive or negative. In other words, loadings of the initial data into a principal component may be negative. Thus, a high value in a principal component may represent a high or low variance in the underlying phenotypes. Because we are interested in identifying genes that only result in high variance, principal components analysis was not appropriate, and so we used a strategy based on clustering instead.

PAM [77] was selected for dimensional reduction because it has several advantages to k-means clustering with respect to our question: (1) resultant medoid cluster centers are real phenotypes from our residuals of standard deviation matrix (rather than difficult-to-interpret linear combinations of residuals of many phenotypes as in k-means); (2) using medoids as cluster centers rather than average cluster centers makes the procedure more robust to outliers; and (3) an “average silhouette” strategy [78] utilized here allows for estimation of the appropriate number of distinct clusters.

To estimate the number of nonredundant clusters, the average silhouette strategy [78] was used: we performed PAM over a range of 20 to 100 clusters and generated silhouette plots for each. We then plotted the average silhouette width versus the number of clusters chosen (Figure S2). Although the average silhouette width peaks around 80 clusters, significant gaps (silhouette widths close to 0) are more likely to appear in silhouette plots when greater than 70 clusters were chosen, suggesting that noninformative clusters are added beyond 70. Thus, we estimated 70 nonredundant clusters. However, using a range of 50 to 80 clusters for PAM to identify phenotypic capacitors results in an extremely similar set of genes (Figure S3).

Using the reduced matrix of 4,718 YKOs by 70 phenotype medoids, we next calculated a single measure of the overall phenotypic variance resulting from a gene's deletion, which we term the phenotypic potential. We generate this score for each YKO by averaging the top 35 (of 70) residuals of standard deviation. The rationale for using 35 of 70 medoids is as follows: (1) A gene deletion that results in a high variance in only one or a few phenotypes does not meet the pleiotropy requirement of our definition of a phenotypic capacitor. Thus, we sought YKOs that have both a large number of high variance phenotypic medoids and high magnitudes in those medoids (i.e., as many medoids should be averaged as possible to capture the overall phenotypic potential). (2) Only 24 YKOs result in high variance (>1 SD) in greater than 35 phenotype medoids, all of which were subsequently identified as phenotypic capacitors. Thus, phenotypic potential scores that rely on greater than 35 medoids are likely to increase noise in the scoring procedure. Although we chose to score 35 phenotype medoids to identify phenotypic capacitors, alternative procedures result in an extremely similar set of genes over a broad range of number of medoids scored (Figure S4).

### Estimation of the number of phenotypic capacitors using the FDR.

First, we generated 100 randomized phenotypic medoid by YKO matrices by permuting elements within each phenotype column of the original matrix. These 100 matrices are then used to calculate phenotypic potentials. We then generated a reference distribution by averaging the top ranking phenotypic potential for each of the 100 trials, the second top ranking phenotypic potential, etc. This reference distribution was used to estimate the expected false positive rate under the null hypothesis. At a given expected false positive rate, the number of true positives was estimated by subtracting the number of false positives (i.e., expected false positive rate × 4,718) from the number of genes in the actual distribution that have a higher phenotypic potential than the reference distribution (all positives). The maximum number of true positives occurs at an expected false positive rate of 0.036 with 333 true positives estimated for 502 positives (a FDR of 34%).

### Cell staining and visualization.

YKO magic marker deletion haploid convertible diploid strains (Open Biosystems) were grown on YPD agar for ∼48 h, spread on GNA (5% D-glucose, 3% Difco nutrient broth, 1% Difco yeast extract, 2% bacto agar) plates and grown for 24 h. A single medium-sized colony was added to 5 ml of sporulation medium (10% potassium acetate, 0.005% zinc acetate + Ura + His + Leu) and incubated at 30 °C for 5–7 d. One to 10 μl of the sporulated cells were spread onto magic media plates (SC-Leu-His-Arg + canavanine + G418) and grown until medium sized colonies appeared (∼20 generations and for no more than 72 h when possible). Single colonies were frozen at this point for later processing. Cell stocks were streaked out on YEPD agar and grown at 30 °C for a maximum of ∼48 h when possible (∼20 additional generations). Cells were subsequently grown overnight in 3 ml YEPD at 30 °C with shaking. Because many of the haploid YKO strains were expected to include heterogeneous morphologies, direct cell counts using a hemocytometer were relied upon rather than optical density readings. Cells were counted, then 1 × 10^8^ cells were added to 20 ml YEPD and grown for 3–3.5 hrs at 30 °C (early logarithmic phase).

Fixation and straining was performed as described in the CalMorph manual with modifications (http://scmd.gi.k.u-tokyo.ac.jp/datamine/calmorph/). Briefly, cells were fixed in 3.7% formaldehyde, 100 mM potassium phosphate, and triply stained for cell-surface manno-protein, actin cytoskeleton, and nuclear DNA using fluorescein isothiocyanate-Con A (Sigma), rhodamine-phalloidin (Molecular Probes), and 4′, 6-diamidino-2-phenylindole (Sigma), respectively. Cells were mounted in vectashield (Vector Laboratories), and visualized by epifluorescent microscopy on a Nikon Eclipse 90i automated microscope using a 100× objective and a Roper 1K CCD camera. For each YKO, a minimum of 40 micrographs was captured to yield a minimum of 200 (and usually in excess of 300) phenotyped cells that are not classified as “complex.” Captured micrographs were analyzed for quantitative morphological traits using the CalMorph software package. For all YKOs processed, ∼50 generations is estimated to have passed from sporulation to fixation and phenotyping: ∼40 generations on agar plates and ∼10 generations in liquid media. Control strains are defined as those knockouts with a phenotypic potential that ranked below 1,000 in the original analysis of the haploid knockout library.

### GO enrichment.

The full GO term hierarchy was downloaded from the GO website on May 11, 2007 (http://www.geneontology.org/). To determine GO process term enrichment, the hierarchy was first trimmed by removing GO terms that did not annotate three or more yeast ORFs. Additionally, the highly annotated process GO terms “physiological process,” “cellular process,” “biological process,” and “cellular physiological process” were removed because they are too general to be meaningful. Trimmed hierarchies resulted in 775 process terms. Significance of GO term enrichment of the 502 putative phenotypic capacitors was calculated using the hypergeometric distribution and Bonferroni corrected using the trimmed hierarchy.

### Network parameters.

The May 1, 2007 release of interaction data (BIOGRID-ORGANISM-Saccharomyces_cerevisiae-2.0.27.tab.txt) was downloaded from the BioGRID (http://www.thebiogrid.org/) [32].

For analysis of physical interactions, two networks were constructed from the BioGRID: (1) every physical interaction from the hand-curated literature citation interaction database was included (including affinity capture-mass spectrometry, affinity capture-western, affinity capture-RNA, cofractionation, colocalization, copurification, fluorescence resonance energy transfer (FRET), two-hybrid, biochemical activity, cocrystal structure, far western, protein–peptide, protein–RNA, reconstituted complex; 5,192 nodes, 70,900 edges); or (2) only interactions from the higher confidence affinity capture-mass spectrometry (3,686 nodes, 47,774 edges). Because nearly every gene has been used as bait for the affinity capture-mass spectrometry network [33,34] and because of the higher consistency and lower rate of false positives compared to other methods, this network might represent the most unbiased view of the PPI network. Thus, this smaller network was used to calculate network properties of genes such as betweenness and clustering coefficient, to control for PPIs when estimating other gene properties such as synthetic lethality and dispensibility, and to estimate the properties of immediate neighbors in the physical interaction network.

All interactions in the BioGRID annotated with “Synthetic Lethality” were used to generate the SLI network (2330 nodes, 18,550 edges). Because the SLI network is incomplete, we hand curated synthetic-lethal entries to create a subset of this network that only includes interactions derived from SGAs where both genes are completely functionally compromised and where the experiment contains ten or more total interactions (1,326 nodes, 11,934 edges). The subset of genes involved in SLIs discovered in SGA experiments was further separated into those genes that have been used as “bait” and those that have not, “prey.” Double knockouts for the 267 bait genes and all other nonessential genes have been performed and thus the complete SLIs for bait genes have likely been discovered; however, double knockouts for the 1,060 prey genes have only been performed with the 267 genes used as bait and thus represent an incomplete, although likely representative, interaction set. Genes without any interactions were excluded from the interaction networks. Betweenness [79] was calculated using the “betweenness” function from the “sna” library in R. The clustering coefficient for each gene was calculated, as described [80].

Essential genes are those genes that were determined to be essential in the systematic deletion project [81] except for the following genes which were deemed nonessential based on subsequent synthetic-lethal analysis: *YJL174W*, *YJR057W*, *YLR103C*, *YOR326W*, *YPL153C*, *YBR234C*, *YDL102W*, *YDL029W*, *YDL017W*, *YDL003W*, *YDR052C* [19,82]. Unless otherwise noted, nonessential genes refers to the 4,718 genes that were knocked-out and phenotyped in the high-dimensional quantitative morphological phenotyping experiment including any identified phenotypic capacitors [17].

### Significance calculation of PPI degree comparison between capacitors and essential genes.

All putative phenotypic capacitors (those with the top 502 phenotypic potential scores) were compared to all essential genes by Wilcoxon-Mann-Whitney test using PPI degrees derived from both the literature citation and mass spectrometry networks (*p* < 2.2 × 10^−16^ and *p* < 5.2 × 10^−13^, respectively). A similar comparison was made using only capacitors in the C1 and C2 classes (*p* < 2.0 × 10^−6^ for the literature citation network, and *p* < 7.8 × 10^−7^ for the mass spectrometry network).

### Genome-wide datasets.

Genome-wide datasets were acquired from the following sources: PPIs and SLIs [32], haploid growth rates [37], heterozygous and homozygous diploid growth rates [38], duplicate and singleton identity [39], ohnolog identity [45], Ks of duplicate pairs [39], mRNA length [43], mRNA abundance [83], protein abundance [84], noise in protein expression in permissive (YEPD) and restrictive (SD) media [46], Ka/Ks [43,85]. The expression similarity between duplicate pairs was acquired from Kafri et al. [39] and was determined using correlations of expression over several reported expression array experiments (Ran Kafri, personal communication). The Ka for duplicates resulting from the whole genome duplication (ohnologs) was calculated as follows: alignments of the S. cerevisiae ohnologs and an ortholog from *Kluyveromyces waltii,* a related species whose divergence precedes the whole genome duplication event, have been previously performed [86]. Ka was calculated from these alignments in PAML [87] using the Yang and Nielsen method [88] with all settings set to default except for “icode,” which was set to 2 to reflect the yeast genome.

### Analysis of covariance to control for the effect of expression level on Ks and Ka.

Because Ks and Ka are correlated with the expression level [43], we performed analyses of covariance (ANCOVAs) to estimate evolution rate differences. ANCOVAs were performed as described with modifications [43]. In one case we used Ks instead of dN, and we used the average mRNA expression level of the two paralogs as the continuous variable. Only duplicate pairs for which the expression level of each paralog has been measured were used for the analysis (Figures S16 and S18).

### FDN score.

Parameters (such as phenotypic potential, dispensability, physical, or synthetic-lethal degree) for the immediate PPI neighbors of a given gene were calculated as follows: first, each gene is given a neighbor score in the parameter by averaging the scores of all of its neighbors. For example, a gene with three neighbors with haploid growth rate scores of 0.5, 0.9, and 1.0 will get a neighbor haploid growth rate score of 0.8. Second, the effect of PPI degree on the neighbor parameter score is removed by plotting the neighbor parameter score for each gene versus its PPI degree, fitting a lowess regression to this plot, and taking residuals of the curve fit as a measure of the neighbor parameter controlled for the number of PPIs. Thus, the gene described above with three neighbors and a neighbor haploid growth rate score of 0.8 will only have a low neighbor growth rate (and a negative residual) if it is low relative to other genes with a similar number of PPIs.

## Supporting Information

Figure S1 Variance Depends on Mean in a Phenotype-Specific Manner Scatter plots of means and standard deviations for 4,718 YKO strains for nine representative phenotypes. Black circles represent individual YKOs and red circles represent the lowess curve fit. Phenotype descriptions and their respective units can be found in the CalMorph manual (http://scmd.gi.k.u-tokyo.ac.jp/datamine/calmorph/).
(11.37 MB PDF)

Figure S2 The Average Silhouette Width Versus the Number of Clusters Chosen for PAM (438 KB PDF)

Figure S3 Phenotypic Capacitor Identification Is Robust to Changes in the Number of Clusters Heatmap of the percent match between the identities of genes with the top 502 phenotypic potentials using a different number of clusters for PAM while holding the number of clusters scored constant at 35.
(268 KB PDF)

Figure S4 Phenotypic Capacitor Identification Is Robust to Changes in the Number of Clusters Scored Heatmap of the percent match between the identities of the top 502 phenotypic potentials using 70 clusters for PAM while changing the number of clusters scored (i.e., the number of top ranked residuals of standard deviation averaged per YKO). Low percent match is only seen when comparing to procedures with a low number of clusters scored. This suggests that genes that cause a high variance in only a few phenotypes are different from those that cause high variance globally.
(618 KB PDF)

Figure S5 Phenotypic Capacitors Are Not Biased toward the Mean Extremes Where the Lowess Curve Fit Might Be Less Accurate Scatter plots of means and standard deviations for 4,718 YKO strains for nine representative phenotypes are shown. YKOs with the top 100 phenotypic potentials are plotted in blue and the lowess curve fit is plotted in red. All other YKOs are plotted in grey. Phenotype descriptions and their respective units can be found in the CalMorph manual (http://scmd.gi.k.u-tokyo.ac.jp/datamine/calmorph/).
(11.41 MB PDF)

Figure S6 Identification of Phenotypic Capacitors Is Robust to General Changes in Procedure As an alternate procedure to identify phenotypic capacitors that does not rely on lowess regression or PAM, we calculated phenotypic potentials using the coefficient of variation (CV) matrix, controlling for confounding effects of differences in mean phenotype by eliminating CVs corresponding to mean outliers (described fully in the Protocol S1). Top ranking genes are similar to those using procedures described in the main text. Plotted is the percent of genes identified as phenotypic capacitors (the top 502 scoring genes using procedures described in the main text) for the top 100, 200, 300, 400, and 500 scoring genes using the alternative procedure.
(169 KB PDF)

Figure S7 The Estimated Number of True Positive Identifications of a Phenotypic Capacitor Are Plotted Versus the Expected False Positive Rate under the Null Hypothesis Described fully in the Materials and Methods.
(565 KB PDF)

Figure S8 Phenotypic Capacitor YKOs Have Highly Variable and Distinct Phenotypes Representative micrographs of control (A and B) and phenotypic capacitor (C and D) strains are shown. Cells are stained with FITC-concanavalin A (green), rhodamine phalloidin (red), and DAPI (blue).
(3.63 MB PDF)

Figure S9 Histograms of the Phenotypic Potentials of Control (Black) and Phenotypic Capacitor (Red) YKOs of Haploid-Converted Heterozygous Diploid Strains Using Sampling Analysis (119 KB PDF)

Figure S10 Phenotypic Capacitors Have More PPIs than GO Matched Genes The average number of physical interactions of phenotypic capacitors in the C1 or C2 classes (blue) versus all nonessential (light grey) or essential (dark grey) genes annotated within a given GO process category using all physical interactions in the literature-curated network of the BioGRID (solid bars) or only affinity-capture mass spectrometry interactions (diagonal stripes). All *p*-values are a comparison to nonessential genes, Wilcoxon-Mann-Whitney test: *, *p* < 0.05; **, *p* < 0.001; ***, *p* < 1 × 10^−5^; ****, *p* < 1 × 10^−10^.
(409 KB PDF)

Figure S11 The Percent of Phenotypic Capacitors in the Set of All Nonessential Genes as a Function of Binned Affinity-Capture Mass Spectroscopy PPI Degree (A), SLI Degree (B), or Haploid Growth Rate (C) (469 KB PDF)

Figure S12 Barplots of Synthetic-Lethal Degree and Growth Rate, Controlled for the Number PPIs The lowess regression residual values for each parameter were calculated and compared (see Protocol S1). Genes that fall into the C1 (dark blue), C2 (blue), or C3 (light blue) classes represent high, mid, and low confidence phenotypic capacitors, respectively. The abbreviations LC, AMS, and SGA refer to networks derived from all literature-citation interactions, affinity-capture mass spectrometry interactions, and SGA interactions, respectively. All *p*-values are a comparison to nonessential genes, Wilcoxon-Mann-Whitney test: *, *p* < 0.05; **, *p* < 0.001; ***, *p* < 1 × 10^−5^; ****, *p* < 1 × 10^−10^.
(263 KB PDF)

Figure S13 Phenotypic Capacitors Have More Synthetic-Lethal Interactions Than GO Matched Genes The average number of SLIs of phenotypic capacitors in the C1 or C2 classes (blue) versus all nonessential (light grey) genes annotated within a given GO process category using all interactions annotated in the literature curated BioGRID network (solid bars), interactions only derived from SGA experiments considering only genes used as bait (diagonal stripes), and interactions only derived from SGA experiments considering only genes not used as bait (horizontal stripes). All *p*-values are a comparison to nonessential genes, Wilcoxon-Mann-Whitney test: *, *p* < 0.05; **, *p* < 0.001; ***, *p* < 1 × 10^−5^; ****, *p* < 1 × 10^−10^.
(564 KB PDF)

Figure S14 Phenotypic Capacitors Are Less Dispensable Than GO Matched Genes The average growth rates of haploid KO (solid bars), heterozygous diploid KO (diagonal stripes), and homozygous diploid KO (horizontal stripes) strains of phenotypic capacitors in the C1 or C2 classes (blue) versus all nonessential (light grey) genes annotated within a given GO process category. All *p*-values are a comparison to nonessential genes, Wilcoxon-Mann-Whitney test: *, *p* < 0.05; **, *p* < 0.001; ***, *p* < 1 × 10^−5^; ****, *p* < 1 ×10^−10^.
(466 KB PDF)

Figure S15 Barplots of All Nonessential Genes (Grey), All Nonessential Singletons (Light Green), Capacitor Singletons (Light Blue), All Nonessential Duplicates (Dark Green), Capacitor Duplicates (Dark Blue), and All Essential Genes (Dark Grey) for Several Genome-Wide Datasets The abbreviations LC, AMS, and SGA refer to networks derived from all literature-citation interactions, affinity-capture mass spectrometry interactions, and SGA interactions, respectively. All other parameters are described in the Materials and Methods. Wilcoxon-Mann-Whitney test: *, *p* < 0.05; **, *p* < 0.001; ***, *p* < 1 × 10^−5^; ****, *p* < 1 × 10^−10^.
(623 KB PDF)

Figure S16 Binary Analysis of the Effect of Duplicate Identity on the Ks between Paralogs (A) Duplicate pairs that contain at least one capacitor (blue) are more ancient than nonessential noncapacitor duplicate pairs (light grey, *p* < 0.01, ANCOVA).
(B) Duplicate pairs that contain at least one essential gene (dark grey) are more ancient than nonessential noncapacitor duplicate pairs (light grey, *p* < 9.05 × 10^−9^, ANCOVA).
(C) Essential duplicate pairs (dark grey) are more ancient than capacitor duplicate pairs (blue, *p* < 1.03 × 10^−8^, ANCOVA).
(728 KB PDF)

Figure S17 Histograms of the Ks between Paralogs of Duplicate Pairs That Contain at Least One Capacitor (Blue) or only Nonessential, Noncapacitor Genes (Grey) (134 KB PDF)

Figure S18 Binary Analysis of the Effect of Duplicate Identity on the Ka between Ohnologs (A) Ohnologs that contain at least one capacitor (blue) are plotted against nonessential noncapacitor ohnologs (light grey, *p* = 0.99, ANCOVA).
(B) Ohnologs that contain at least one essential gene (dark grey) are plotted against nonessential noncapacitor ohnologs (light grey, *p* < 0.02, ANCOVA).
(C) Essential ohnologs (dark grey) are plotted against capacitor ohnologs (blue, *p* < 0.07, ANCOVA).
(620 KB PDF)

Figure S19 Barplots of the First-Degree PPI Neighbors of a Gene Controlled for the Degree of That Gene for Several Genome-Wide Datasets (FDN Score, see Materials and Methods) Colors are nonessential genes (grey), all nonessential singletons (light green), capacitor singletons (light blue), all nonessential duplicates (dark green), capacitor duplicates (dark blue), and all essential genes (dark grey). The abbreviations LC and AMS refer to networks derived from all literature citation interactions and affinity-capture mass spectrometry interactions, respectively. All other parameters are described in the methods. Wilcoxon-Mann-Whitney test: *, *p* <0.05.
(827 KB PDF)

Protocol S1 Supplementary Methods (28 KB PDF)

Table S1 S. cerevisiae Genes Ranked by Phenotypic Potential (893 KB TXT)

Table S2 GO Process Terms with Significant Enrichment of Phenotypic Capacitors (47 KB TXT)

Table S3 GO Process Terms with Significant Enrichment of Singleton or Duplicate Phenotypic Capacitors (18 KB TXT)

## Abbreviations

GCR: gross chromosomal rearrangement
GO: Gene Ontology
FDN: first-degree neighbor
FDR: false discovery rate
Ka: nonsynonymous substitution rate
Ks: synonymous substitution rate
PAM: partitioning around medoids
PPI: protein–protein interaction
SGA: synthetic genetic array
SLI: synthetic lethal interaction
YKO: yeast single-gene knockout
