# Supplementary material for: Network Hubs Buffer Environmental Variation in Saccharomyces cerevisiae
Source: PLoS Biol. 2008 Nov 4;6(11):e264. doi: 10.1371/journal.pbio.0060264 (PMC2577700; doi:10.1371/journal.pbio.0060264)
Supplement: Supplementary file 1 [file pbio.0060264.sd001.pdf]

Protocol S1

Alternate method to identify phenotypic capacitors. As an alternate procedure to identify phenotypic capacitors that does not rely on lowess regression or partitioning around medoids, we calculated the coefficient of variation (CV) of each YKO for 220 phenotypes and standardized to weight each phenotype equally. Thus, we generated a CV matrix of 4718 YKOs by 220 phenotypes. Because we wished not to confound changes in variance with changes in mean phenotype [1], and because for our data the CV alone does not sufficiently control for variation in the mean, we next removed any values in the CV matrix where the mean phenotype value for that YKO differed by greater than 2 standard deviations from the mean phenotype value for all YKOs. Similar to methods described in the main text, we next generated a single score for the overall variance by ranking the CVs for each YKO and averaging the top 70 (out of a possible 220). YKOs with top ranking scores were extremely similar to those identified using procedures described in the main text (Figure S6). Similar results are also found if, prior to calculating phenotypic potential scores, the PAM clustering procedure is applied to the 4718 X 220 CV matrix from which elements corresponding to mean outliers had been removed (data not shown).

Regressions to control for physical interaction degree. Because correlations might exist between the physical interaction of a gene and other parameters (such as synthetic-lethal degree, dispensability, betweenness, and clustering coefficient), we controlled for physical-interaction degree on these parameters through lowess regression. Similar to the regressions to control variance for differences in mean described in the results, the physical-interaction was first plotted versus the parameter of interest degree for all non-essential genes. Second, a lowess regression was fit and

residuals calculated. These residuals were then used in subsequent statistical comparisons (Figure S11).

Spontaneous mutation and gross chromosomal rearrangement frequency estimation. The spontaneous mutation rate in *S. cerevisiae* is estimated to be ~0.003 per cell per DNA replication based on mutation accumulation experiments [2]. Genes that, when mutated, cause a drastic increase in this rate are relatively well defined [3]. We identify two of these genes in our original screen for phenotypic capacitors (RAD27 and MSH2). Repeating the phenotyping of RAD27 using the haploid convertible diploid strain after ~50 generations of haploid growth resulted in low phenotypic potential, which suggests that mutation accumulation in the original haploid-mutant strain had caused the observed phenotypic variability.

Many other capacitors have been reported to cause the accumulation of gross chromosomal rearrangements (GCRs), such as translocations, deletion of a chromosome arm, interstitial deletions or inversions [4,5]. This suggests that these knockouts might be causing genotypic heterogeneity and thereby phenotypic heterogeneity by mechanisms other than decanalization. For example, the homologous recombination module [6] is composed of eight genes, seven of which were identified as capacitors in our initial screen. We re-phenotyped five of these YKOs and four resulted in higher phenotypic potentials than any of the 50 control strains. To roughly estimate the number of GCRs possible in our re-phenotyped strains, we use a conservative estimate of 100x the maximal break rate of chromosome V observed for any capacitor (2.3×10^{-7} per generation for rad50Δ) [4]. Assuming GCRs do not alter the growth rate, after ~50 generations of growth, the estimated frequency of cells with GCRs would be approximately $(2 \times 10^{-7}) \times (100) \times (50) = 0.001$. Thus, ~1 in 1000 phenotyped cells could contain a GCR. Because we phenotype, on average ~300-400 cells per strain, it is

extremely unlikely that cells containing a GCR would have a measurable effect on the variances measured. Thus, it is unlikely that GCR events explain the high phenotypic variance in these capacitor YKOs.

Ty1 transposition rate and phenotypic potential.

Increased Ty1 transposition rate is neither necessary nor sufficient for high phenotypic potential. As described in the main text, we focused on the eight genes in the homologous recombination module [7] and found YKOs that cause both high phenotypic potentials and increased Ty1 transposition rates (RAD50, RAD51, RAD54, RAD55, RAD57), high phenotypic potentials but normal or decreased Ty1 transposition rates (XRS2), and low phenotypic potentials but increased Ty1 transposition rates (MRE11) [8,9,10].

References:

1. Dworkin I (2005) Canalization, cryptic variation, and developmental buffering: a critical examination and analytical perspective: Elsevier. 132-144 p.

2. Drake JW (1991) A constant rate of spontaneous mutation in DNA-based microbes. Proc Natl Acad Sci U S A 88: 7160-7164.

3. Kolodner RD, Marsischky GT (1999) Eukaryotic DNA mismatch repair. Curr Opin Genet Dev 9: 89-96.

4. Myung K, Chen C, Kolodner RD (2001) Multiple pathways cooperate in the suppression of genome instability in Saccharomyces cerevisiae. Nature 411: 1073-1076.

5. Chen C, Kolodner RD (1999) Gross chromosomal rearrangements in Saccharomyces cerevisiae replication and recombination defective mutants. Nat Genet 23: 81-85.

6. Pan X, Ye P, Yuan DS, Wang X, Bader JS, et al. (2006) A DNA integrity network in the yeast Saccharomyces cerevisiae. Cell 124: 1069-1081.

7. Pan X, Ye P, Yuan DS, Wang X, Bader JS, et al. (2006) A DNA Integrity Network in the Yeast Saccharomyces cerevisiae. Cell 124: 1069-1081.

8. Griffith JL, Coleman LE, Raymond AS, Goodson SG, Pittard WS, et al. (2003) Functional genomics reveals relationships between the retrovirus-like Ty1 element and its host Saccharomyces cerevisiae. Genetics 164: 867-879.

9. Nyswaner KM, Checkley MA, Yi M, Stephens RM, Garfinkel DJ (2008) Chromatin-associated genes protect the yeast genome from Ty1 insertional mutagenesis. Genetics 178: 197-214.

10. Scholes DT, Banerjee M, Bowen B, Curcio MJ (2001) Multiple regulators of Ty1 transposition in Saccharomyces cerevisiae have conserved roles in genome maintenance. Genetics 159: 1449-1465.
