# Supplementary material for: Network Hubs Buffer Environmental Variation in Saccharomyces cerevisiae
Source: PLoS Biol. 2008 Nov 4;6(11):e264. doi: 10.1371/journal.pbio.0060264 (PMC2577700; doi:10.1371/journal.pbio.0060264)
Supplement: Supplementary file 2 [file pbio.0060264.sg001.pdf]

Figure S1

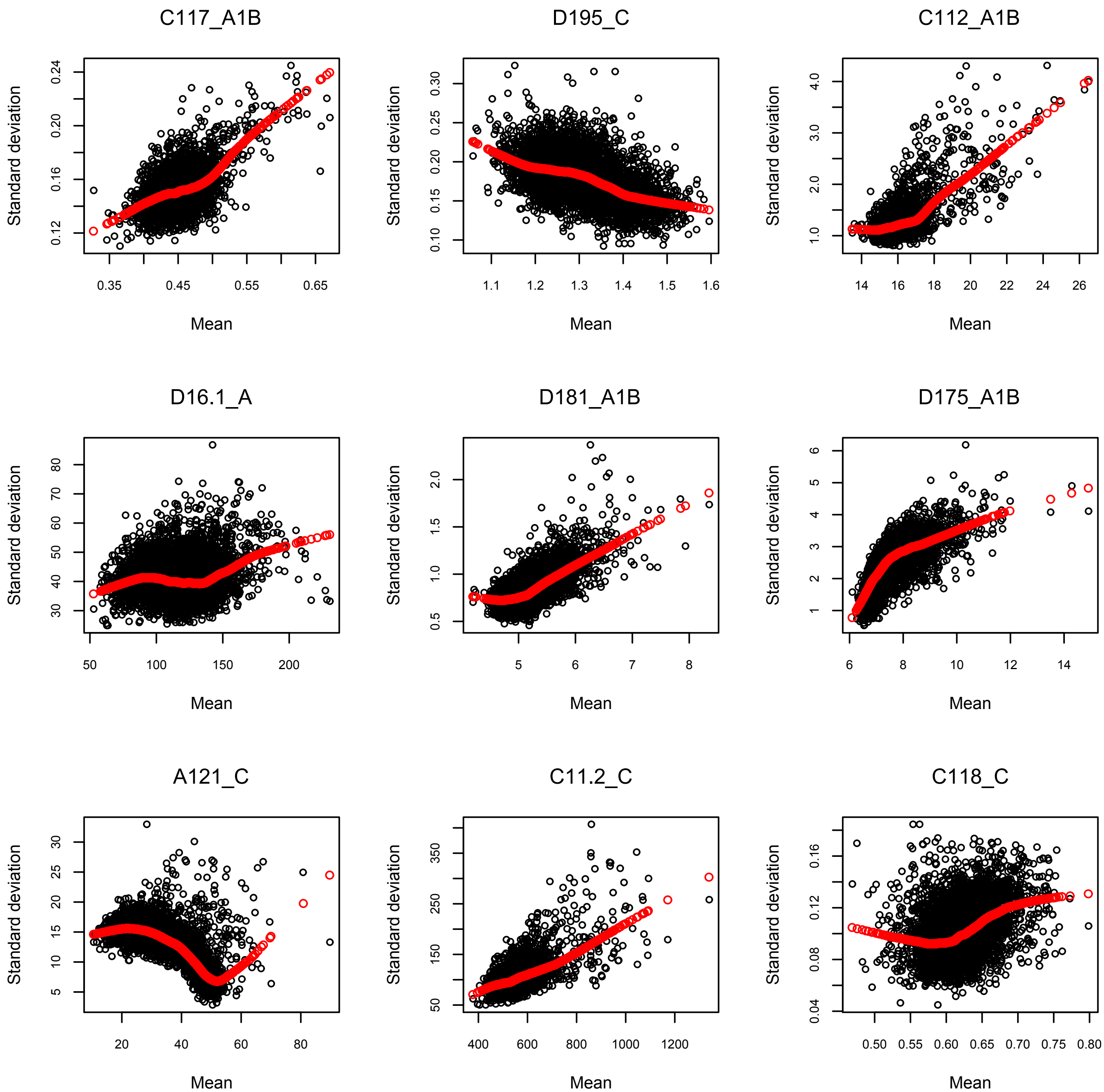
C117_A1B
Standard deviation
0.12 0.16 0.20 0.24
0.35 0.45 0.55 0.65
Mean
D195_C
Standard deviation
0.10 0.15 0.20 0.25 0.30
1.1 1.2 1.3 1.4 1.5 1.6
Mean
C112_A1B
Standard deviation
1.0 2.0 3.0 4.0
14 16 18 20 22 24 26
Mean
D16.1_A
Standard deviation
30 40 50 60 70 80
50 100 150 200
Mean
D181_A1B
Standard deviation
0.5 1.0 1.5 2.0
5 6 7 8
Mean
D175_A1B
Standard deviation
1 2 3 4 5 6
6 8 10 12 14
Mean
A121_C
Standard deviation
5 10 15 20 25 30
20 40 60 80
Mean
C11.2_C
Standard deviation
50 150 250 350
400 600 800 1000 1200
Mean
C118_C
Standard deviation
0.04 0.08 0.12 0.16
0.50 0.55 0.60 0.65 0.70 0.75 0.80
Mean
