# Supplementary material for: Network Hubs Buffer Environmental Variation in Saccharomyces cerevisiae
Source: PLoS Biol. 2008 Nov 4;6(11):e264. doi: 10.1371/journal.pbio.0060264 (PMC2577700; doi:10.1371/journal.pbio.0060264)
Supplement: Supplementary file 3 [file pbio.0060264.sg002.pdf]

Figure S2

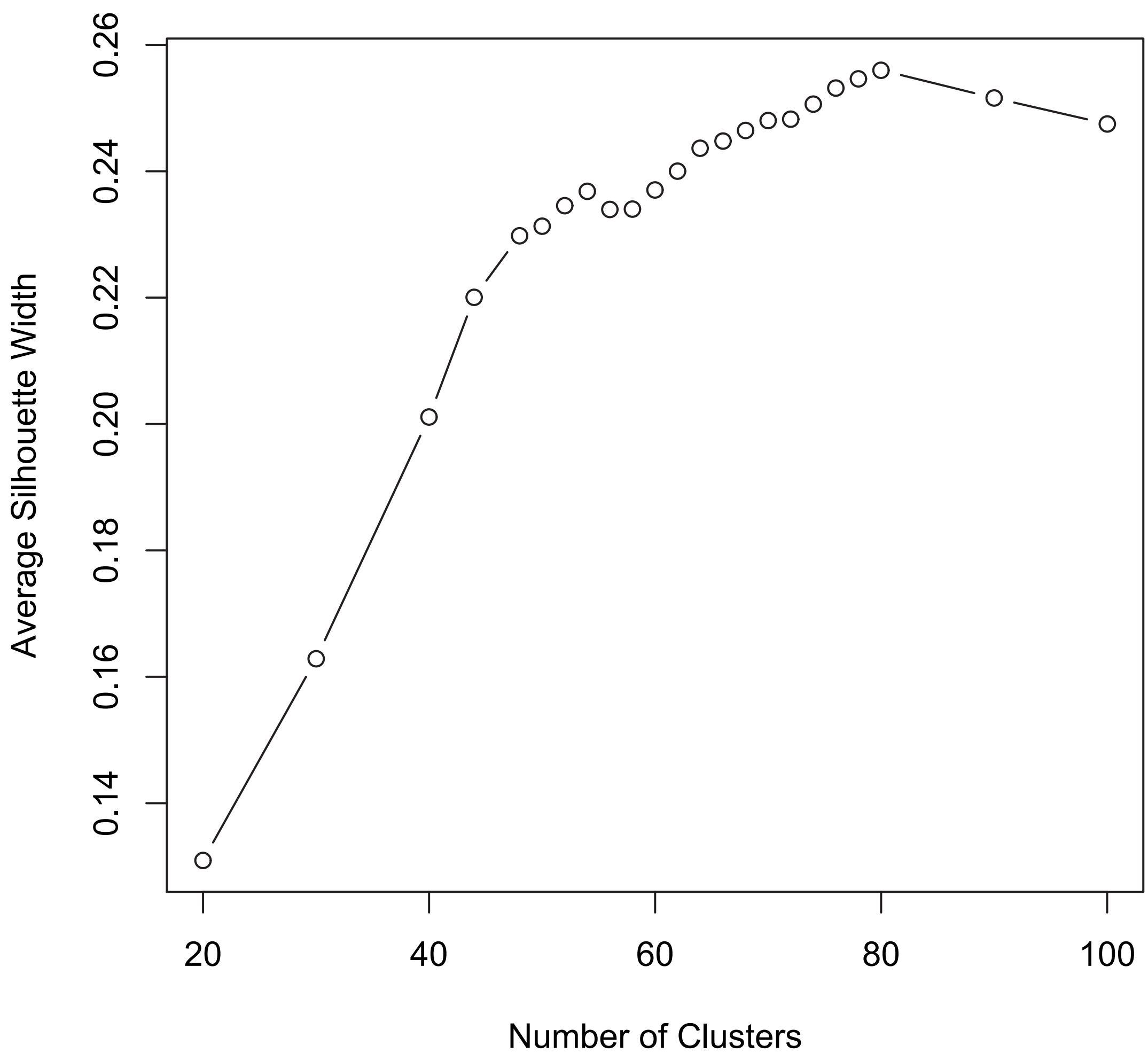

Average Silhouette Width
0.14
0.16
0.18
0.20
0.22
0.24
0.26
20
40
60
80
100
Number of Clusters
