# Supplementary material for: Network Hubs Buffer Environmental Variation in Saccharomyces cerevisiae
Source: PLoS Biol. 2008 Nov 4;6(11):e264. doi: 10.1371/journal.pbio.0060264 (PMC2577700; doi:10.1371/journal.pbio.0060264)
Supplement: Supplementary file 7 [file pbio.0060264.sg006.pdf]

Figure S6

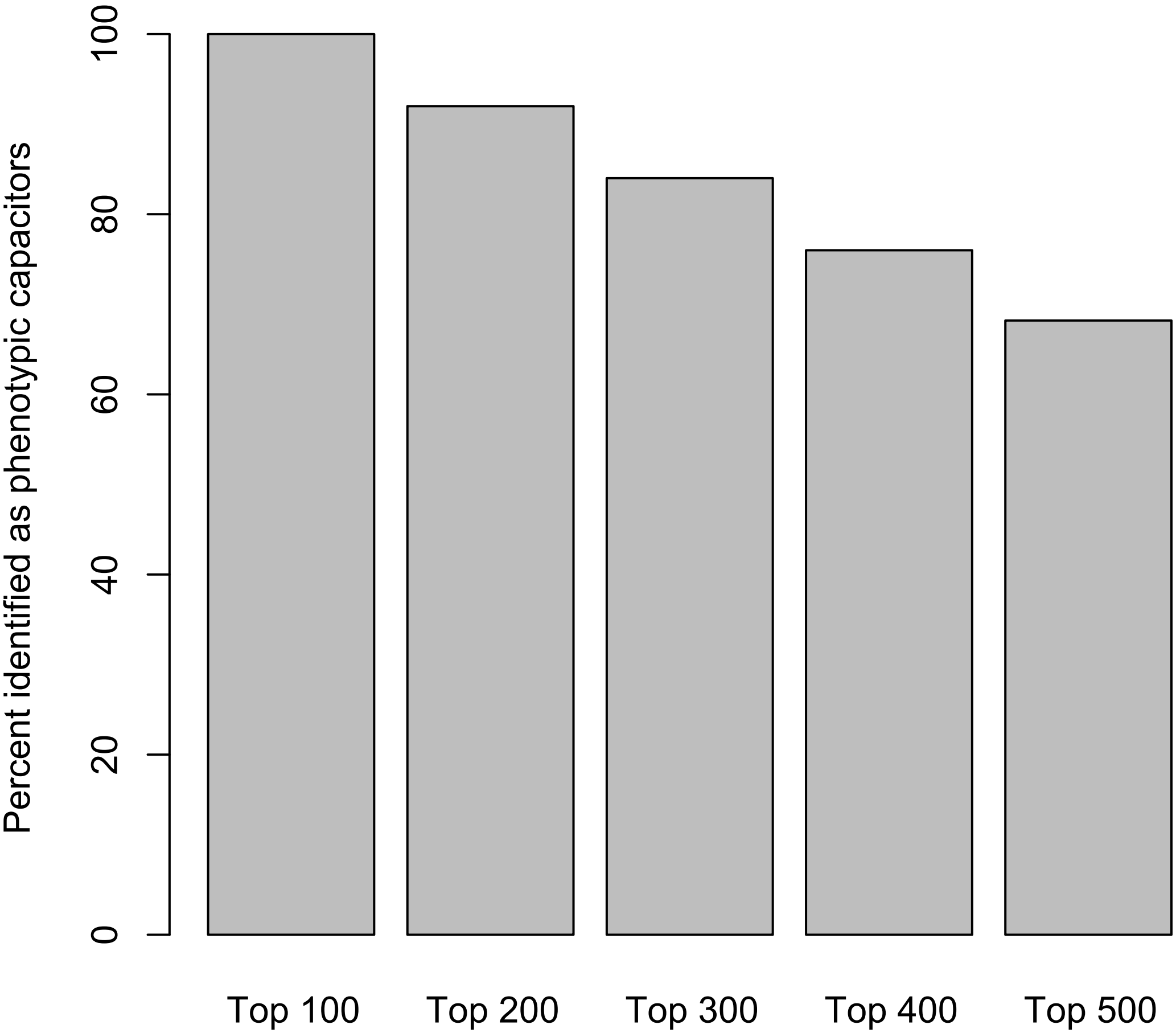
Percent identified as phenotypic capacitors
0
20
40
60
80
100
Top 100
Top 200
Top 300
Top 400
Top 500
