# Supplementary material for: Network Hubs Buffer Environmental Variation in Saccharomyces cerevisiae
Source: PLoS Biol. 2008 Nov 4;6(11):e264. doi: 10.1371/journal.pbio.0060264 (PMC2577700; doi:10.1371/journal.pbio.0060264)
Supplement: Supplementary file 8 [file pbio.0060264.sg007.pdf]

Figure S7

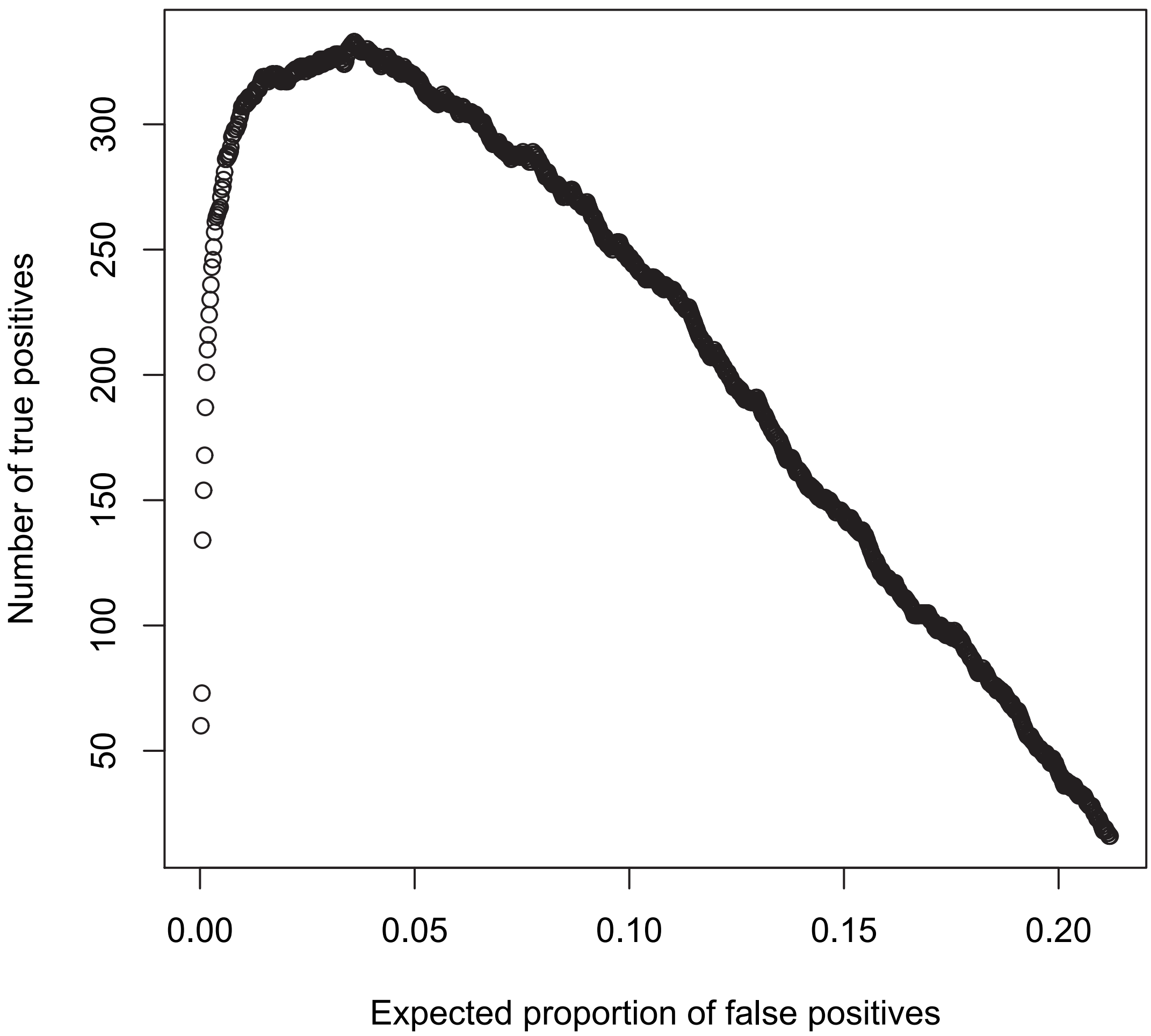

Number of true positives
300
250
200
150
100
50
0.00
0.05
0.10
0.15
0.20
Expected proportion of false positives
