# Supplementary material for: Network Hubs Buffer Environmental Variation in Saccharomyces cerevisiae
Source: PLoS Biol. 2008 Nov 4;6(11):e264. doi: 10.1371/journal.pbio.0060264 (PMC2577700; doi:10.1371/journal.pbio.0060264)
Supplement: Supplementary file 9 [file pbio.0060264.sg008.pdf]

A

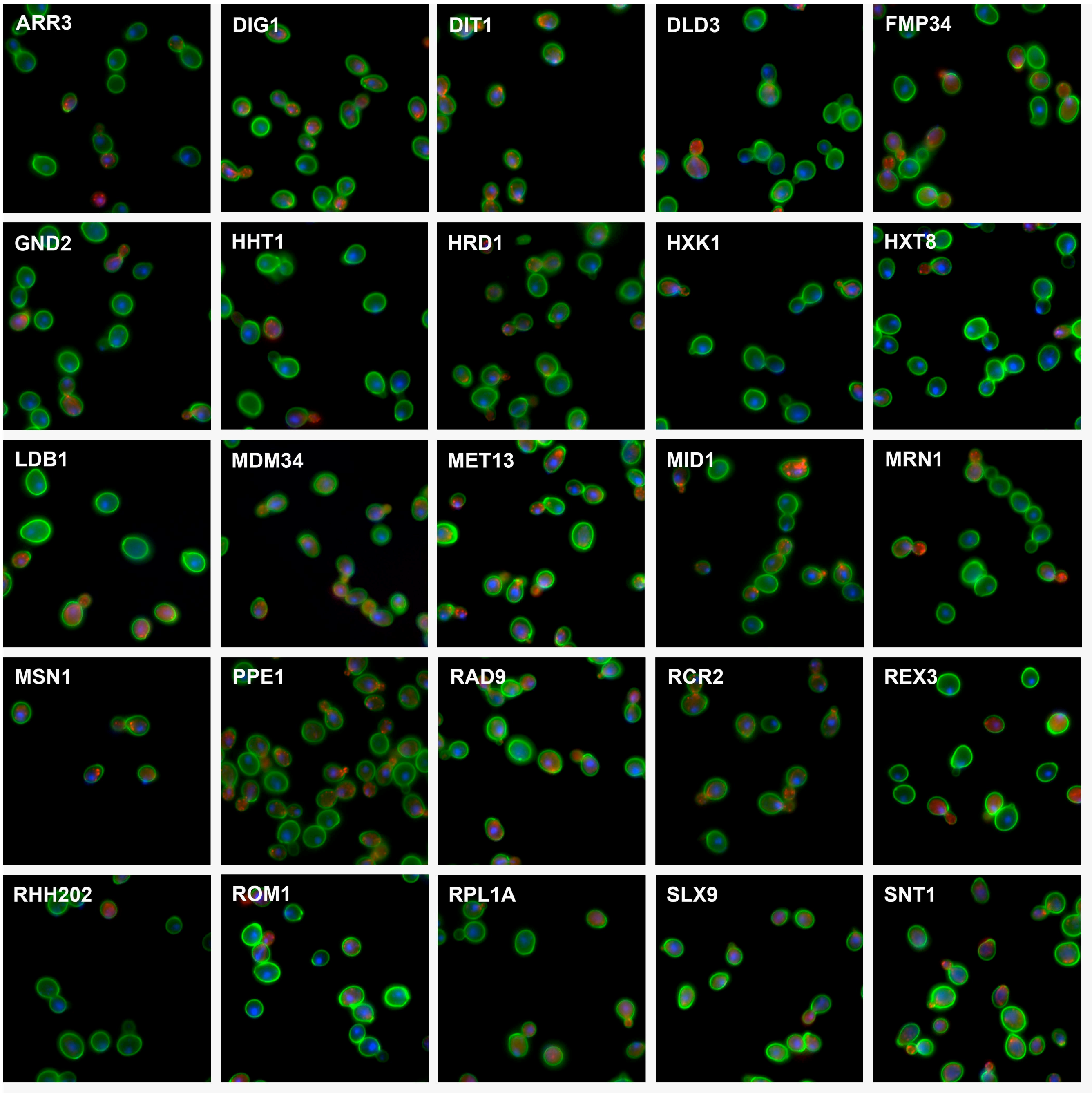
ARR3
DIG1
DIT1
DLD3
FMP34
GND2
HHT1
HRD1
HXK1
HXT8
LDB1
MDM34
MET13
MID1
MRN1
MSN1
PPE1
RAD9
RCR2
REX3
RHH202
ROM1
RPL1A
SLX9
SNT1

B

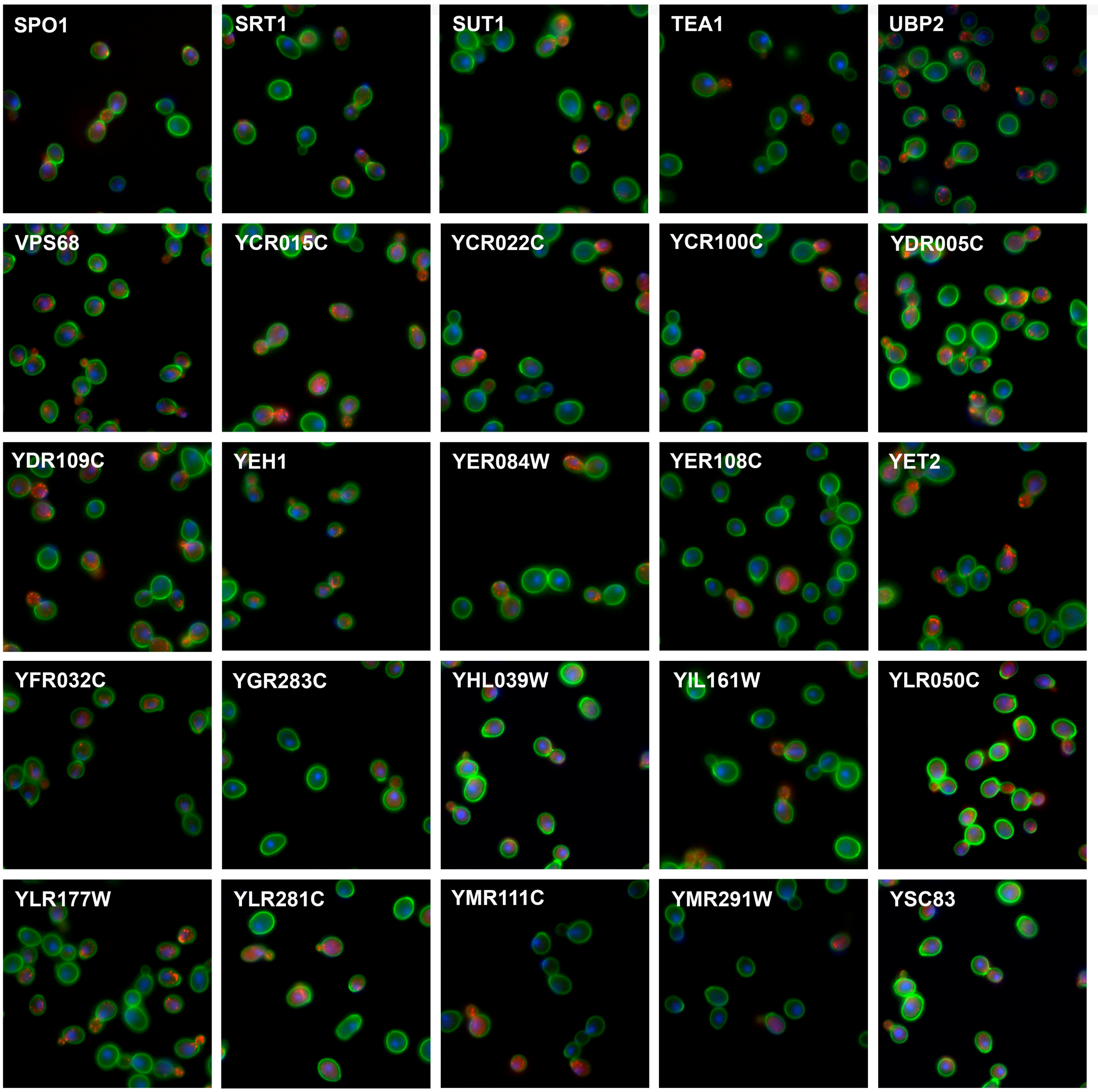
SPO1
SRT1
SUT1
TEA1
UBP2
VPS68
YCR015C
YCR022C
YCR100C
YDR005C
YDR109C
YEH1
YER084W
YER108C
YET2
YFR032C
YGR283C
YHL039W
YIL161W
YLR050C
YLR177W
YLR281C
YMR111C
YMR291W
YSC83

C

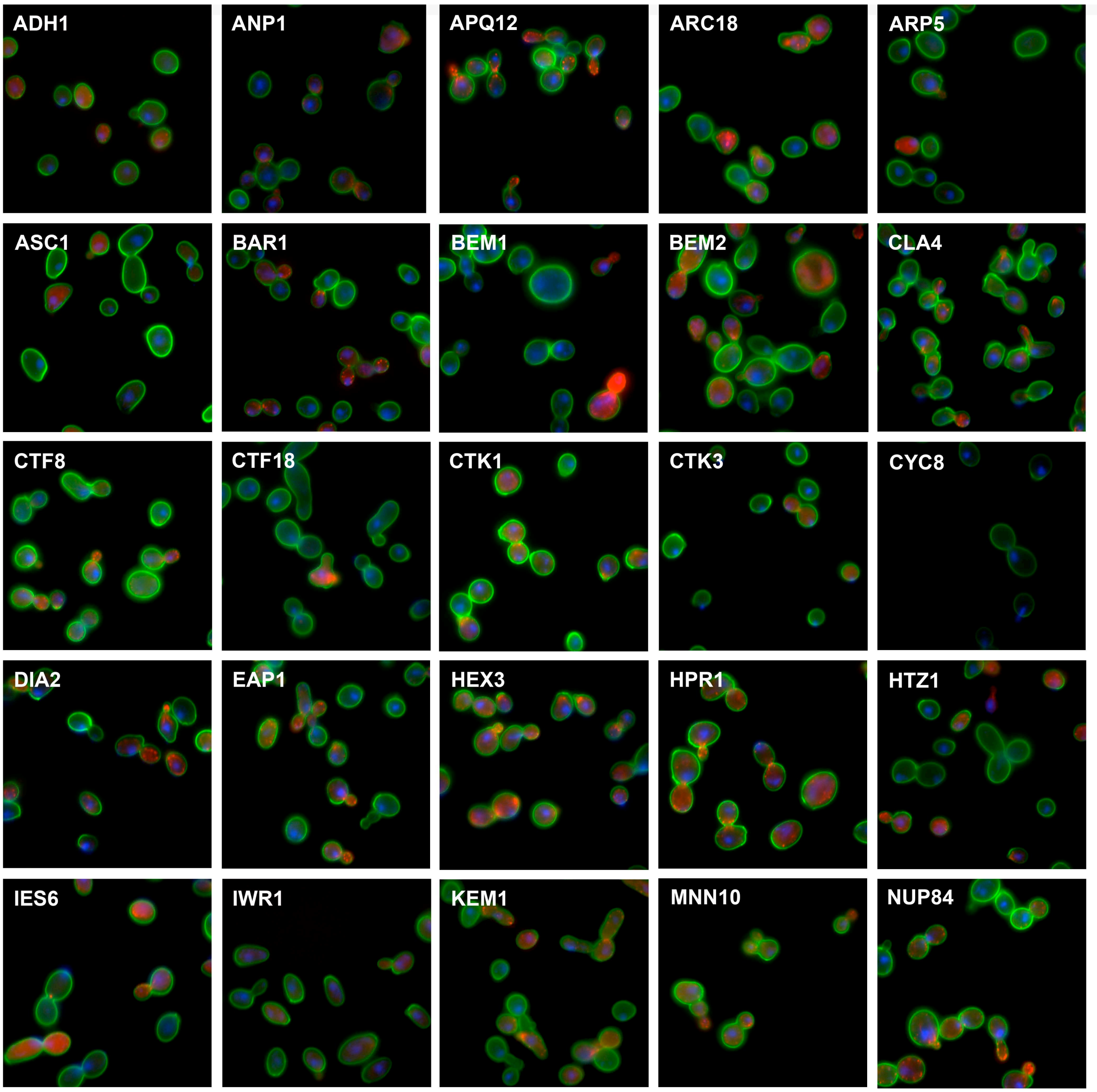
ADH1
ANP1
APQ12
ARC18
ARP5
ASC1
BAR1
BEM1
BEM2
CLA4
CTF8
CTF18
CTK1
CTK3
CYC8
DIA2
EAP1
HEX3
HPR1
HTZ1
IES6
IWR1
KEM1
MNN10
NUP84

D

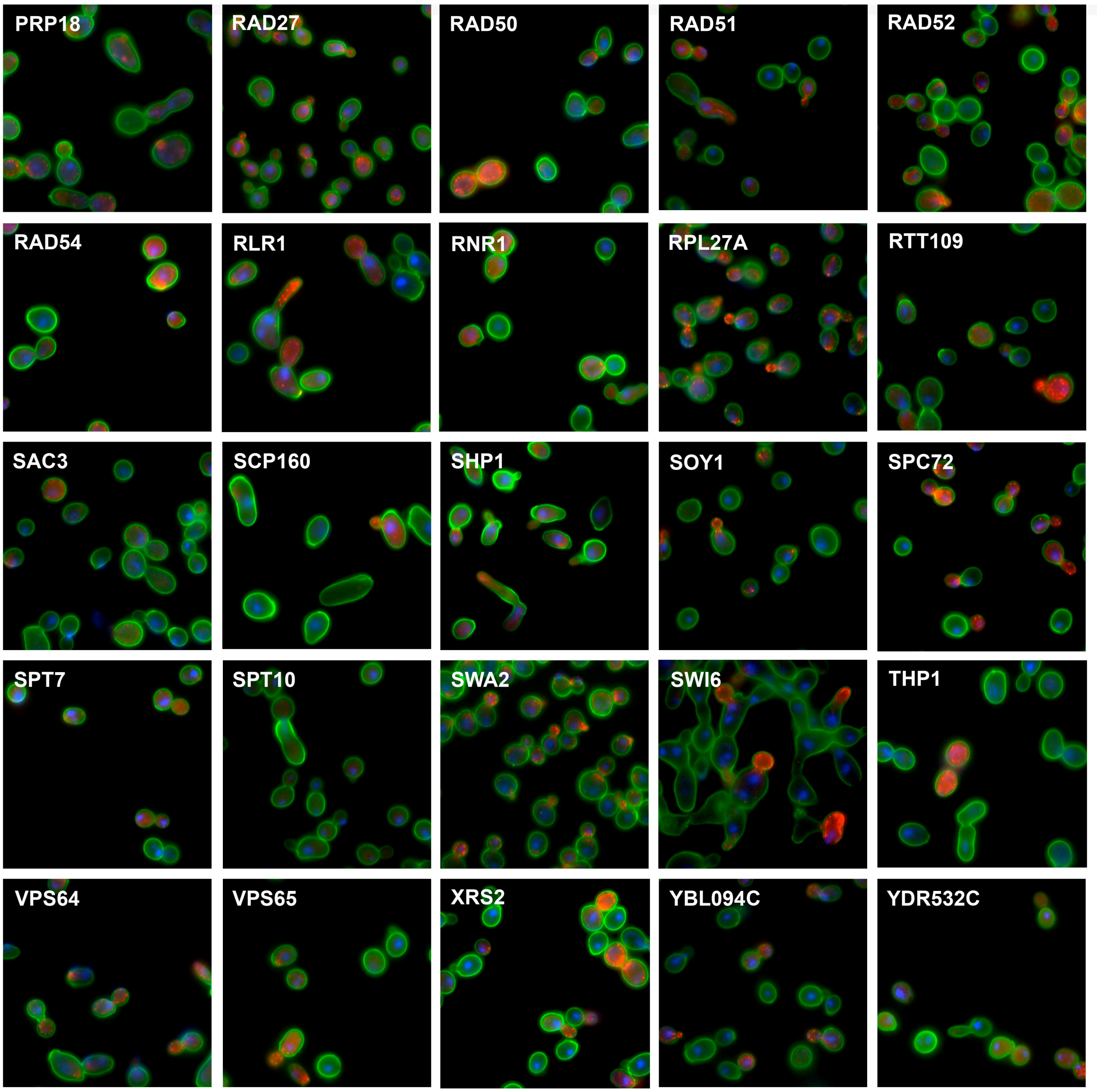
PRP18
RAD27
RAD50
RAD51
RAD52
RAD54
RLR1
RNR1
RPL27A
RTT109
SAC3
SCP160
SHP1
SOY1
SPC72
SPT7
SPT10
SWA2
SWI6
THP1
VPS64
VPS65
XRS2
YBL094C
YDR532C
