# Supplementary material for: Network Hubs Buffer Environmental Variation in Saccharomyces cerevisiae
Source: PLoS Biol. 2008 Nov 4;6(11):e264. doi: 10.1371/journal.pbio.0060264 (PMC2577700; doi:10.1371/journal.pbio.0060264)
Supplement: Supplementary file 12 [file pbio.0060264.sg011.pdf]

Figure S11

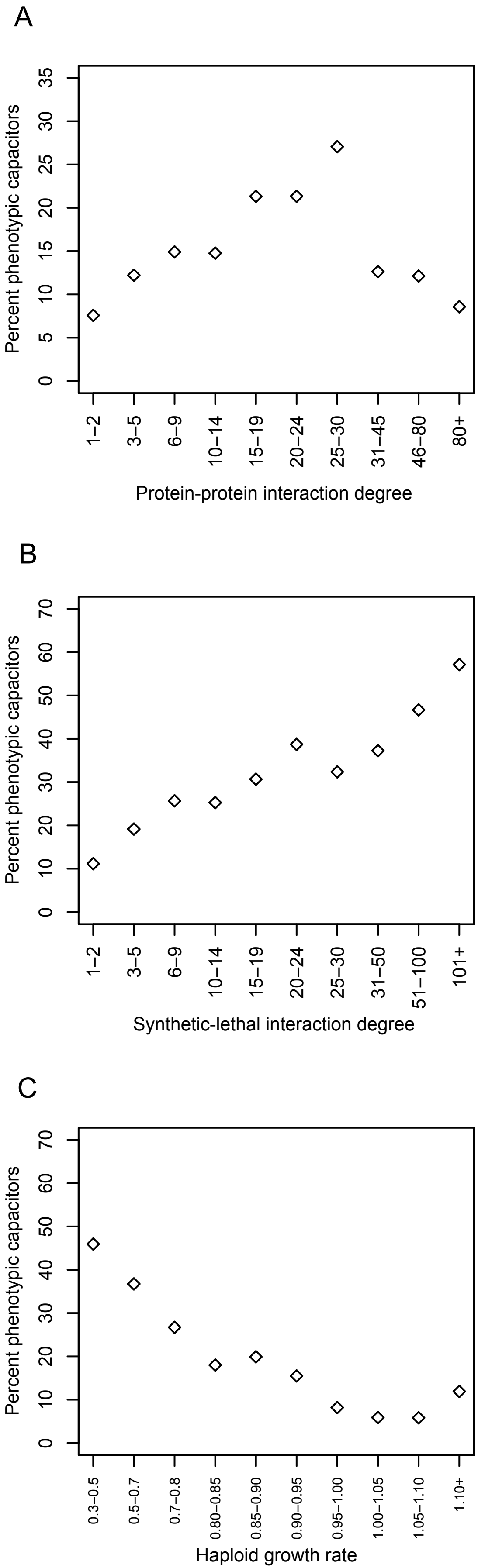
A
Percent phenotypic capacitors
0
5
10
15
20
25
30
35
1–2
3–5
6–9
10–14
15–19
20–24
25–30
31–45
46–80
80+
Protein-protein interaction degree
B
Percent phenotypic capacitors
0
10
20
30
40
50
60
70
1–2
3–5
6–9
10–14
15–19
20–24
25–30
31–50
51–100
101+
Synthetic-lethal interaction degree
C
Percent phenotypic capacitors
0
10
20
30
40
50
60
70
0.3–0.5
0.5–0.7
0.7–0.8
0.80–0.85
0.85–0.90
0.90–0.95
0.95–1.00
1.00–1.05
1.05–1.10
1.10+
Haploid growth rate
