# Supplementary material for: Network Hubs Buffer Environmental Variation in Saccharomyces cerevisiae
Source: PLoS Biol. 2008 Nov 4;6(11):e264. doi: 10.1371/journal.pbio.0060264 (PMC2577700; doi:10.1371/journal.pbio.0060264)
Supplement: Supplementary file 13 [file pbio.0060264.sg012.pdf]

Figure S12

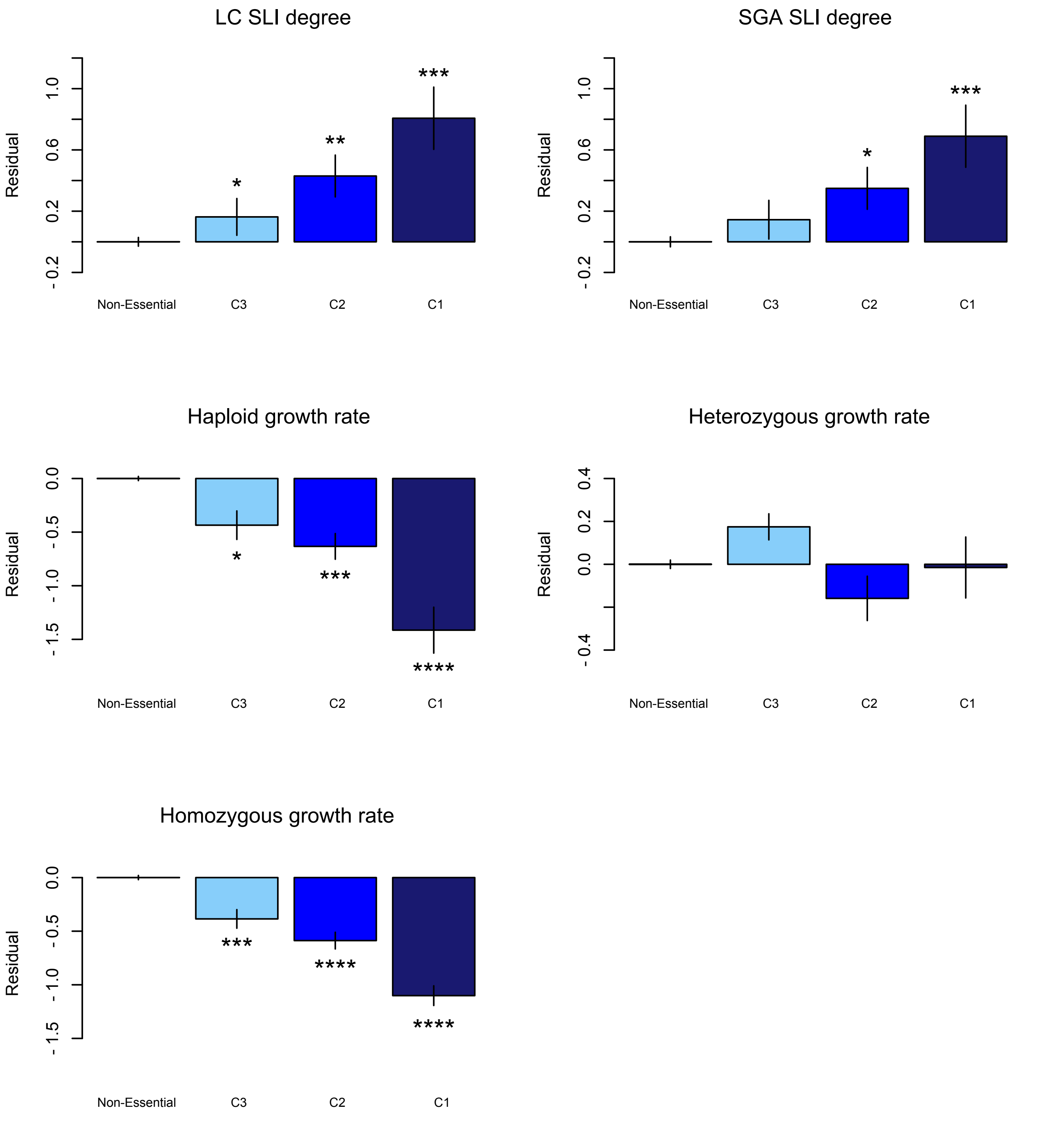
LC SLI degree
SGA SLI degree
Haploid growth rate
Heterozygous growth rate
Homozygous growth rate
Residual
Non-Essential
C3
C2
C1
*
**
***
****
