# Supplementary material for: Network Hubs Buffer Environmental Variation in Saccharomyces cerevisiae
Source: PLoS Biol. 2008 Nov 4;6(11):e264. doi: 10.1371/journal.pbio.0060264 (PMC2577700; doi:10.1371/journal.pbio.0060264)
Supplement: Supplementary file 14 [file pbio.0060264.sg013.pdf]

Figure S13

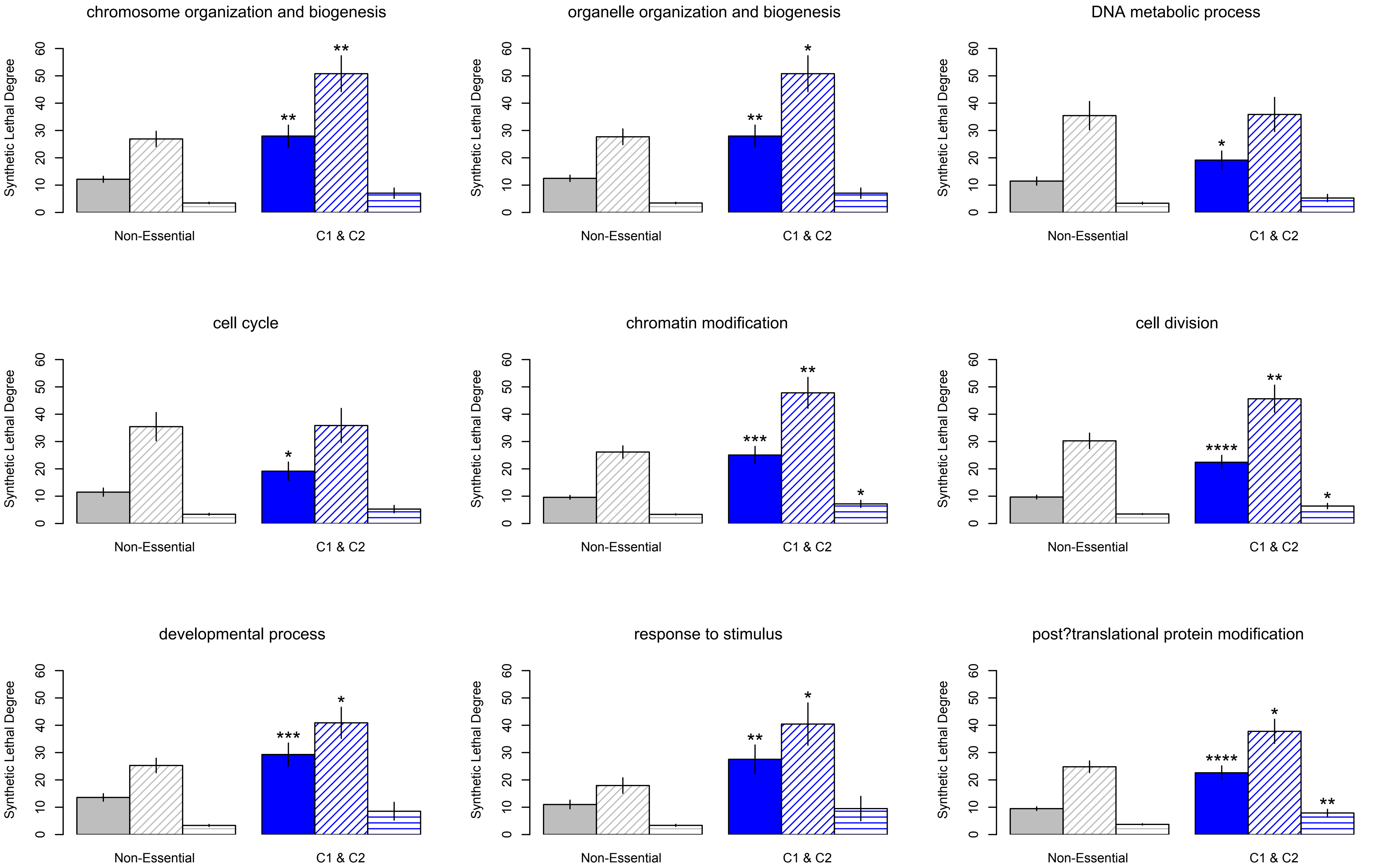
chromosome organization and biogenesis
organelle organization and biogenesis
DNA metabolic process
cell cycle
chromatin modification
cell division
developmental process
response to stimulus
post?translational protein modification
Synthetic Lethal Degree
0
10
20
30
40
50
60
Non-Essential
C1 & C2
*
**
***
****
