# Supplementary material for: Network Hubs Buffer Environmental Variation in Saccharomyces cerevisiae
Source: PLoS Biol. 2008 Nov 4;6(11):e264. doi: 10.1371/journal.pbio.0060264 (PMC2577700; doi:10.1371/journal.pbio.0060264)
Supplement: Supplementary file 15 [file pbio.0060264.sg014.pdf]

Figure S14

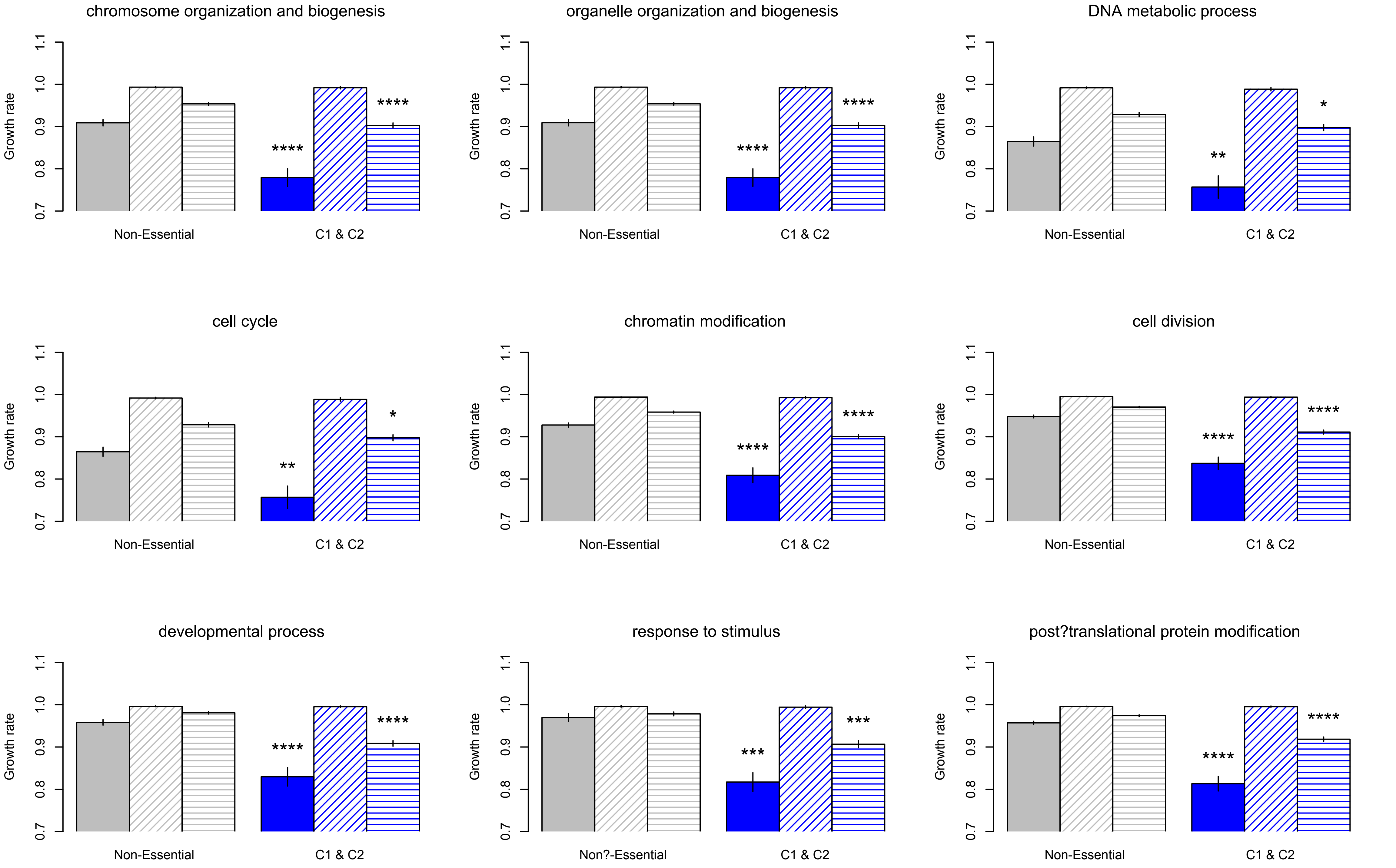
chromosome organization and biogenesis
organelle organization and biogenesis
DNA metabolic process
cell cycle
chromatin modification
cell division
developmental process
response to stimulus
post?translational protein modification
Growth rate
0.7
0.8
0.9
1.0
1.1
Non-Essential
Non?-Essential
C1 & C2
****
***
**
*
