# Supplementary material for: Network Hubs Buffer Environmental Variation in Saccharomyces cerevisiae
Source: PLoS Biol. 2008 Nov 4;6(11):e264. doi: 10.1371/journal.pbio.0060264 (PMC2577700; doi:10.1371/journal.pbio.0060264)
Supplement: Supplementary file 16 [file pbio.0060264.sg015.pdf]

Figure S15

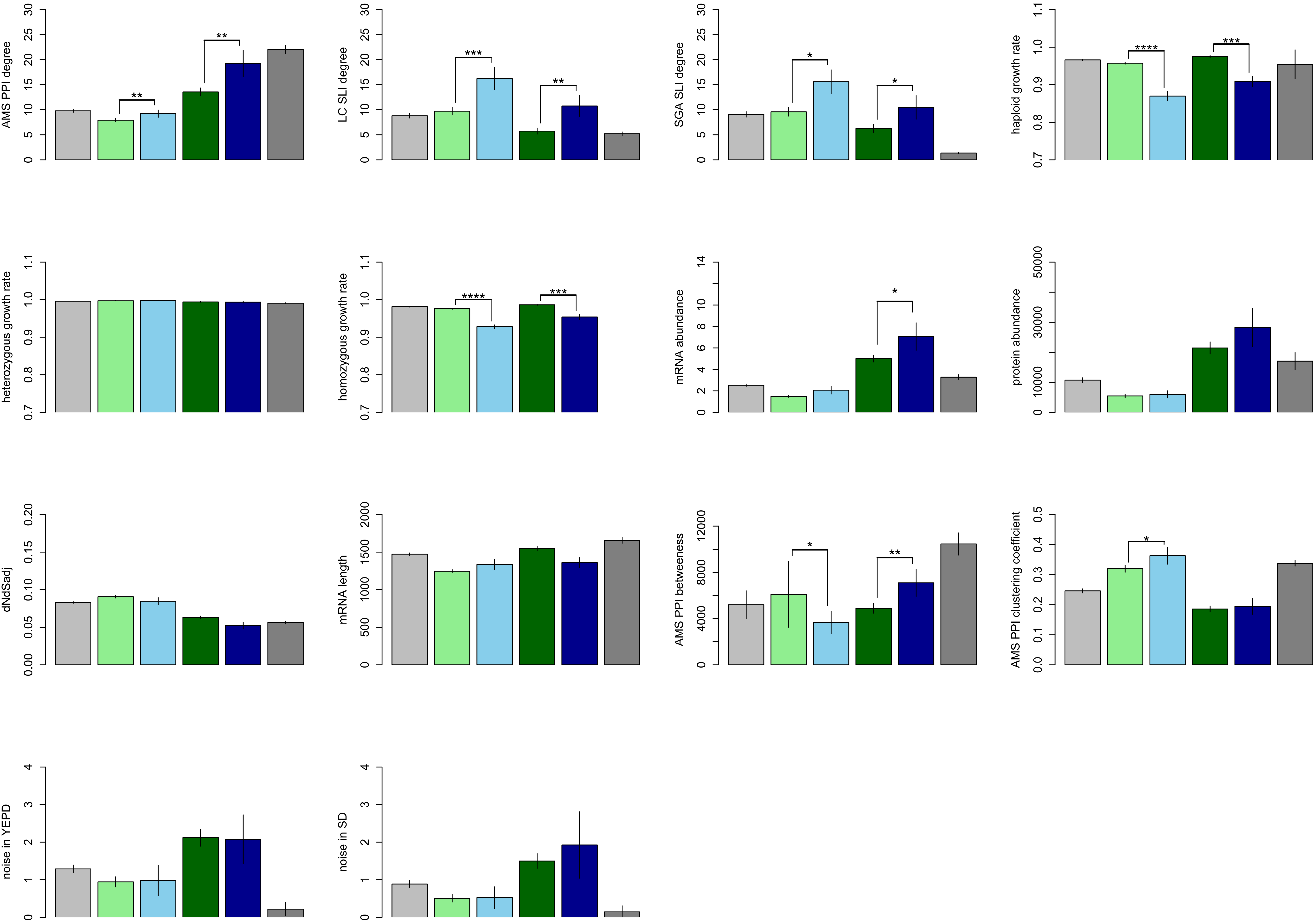
AMS PPI degree
LC SLI degree
SGA SLI degree
haploid growth rate
heterozygous growth rate
homozygous growth rate
mRNA abundance
protein abundance
dNdSadj
mRNA length
AMS PPI betweeness
AMS PPI clustering coefficient
noise in YEPD
noise in SD
