# Supplementary material for: Network Hubs Buffer Environmental Variation in Saccharomyces cerevisiae
Source: PLoS Biol. 2008 Nov 4;6(11):e264. doi: 10.1371/journal.pbio.0060264 (PMC2577700; doi:10.1371/journal.pbio.0060264)
Supplement: Supplementary file 20 [file pbio.0060264.sg019.pdf]

Figure S19

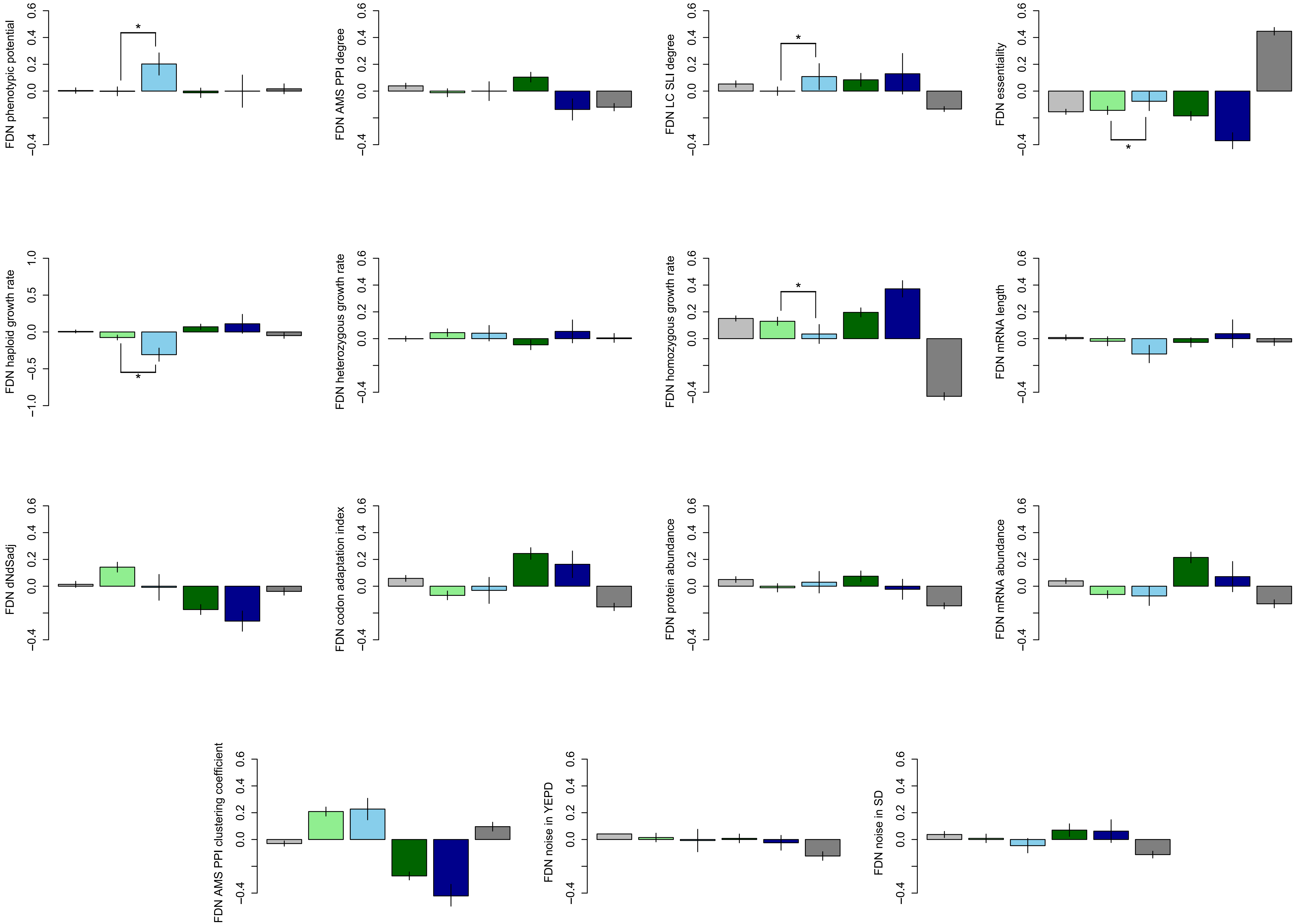

FDN phenotypic potential
FDN AMS PPI degree
FDN LC SLI degree
FDN essentiality
FDN haploid growth rate
FDN heterozygous growth rate
FDN homozygous growth rate
FDN mRNA length
FDN dNdSadj
FDN codon adaptation index
FDN protein abundance
FDN mRNA abundance
FDN AMS PPI clustering coefficient
FDN noise in YEPD
FDN noise in SD
